# Screening of Genes Related to Early and Late Flowering in Tree Peony Based on Bulked Segregant RNA Sequencing and Verification by Quantitative Real-Time PCR

**DOI:** 10.3390/molecules23030689

**Published:** 2018-03-19

**Authors:** Xiaogai Hou, Qi Guo, Weiqiang Wei, Lili Guo, Dalong Guo, Lin Zhang

**Affiliations:** 1 College of Agriculture, Henan University of Science & Technology, 263 Kaiyuan Avenue, Luoyang 471023, China; Guoqi0529@126.com (Q.G.); 15136371676@163.com (W.W.); guolili0928@126.com (L.G.); ayzhanglin@126.com (L.Z.); 2 College of Biological Sciences and Technology, Beijing Forestry University, Beijing 100083, China; 3 College of Forestry, Henan University of Science & Technology, 263 Kaiyuan Avenue, Luoyang 471023, China; grapeguo@126.com

**Keywords:** tree peony, BSR-Seq, flowering time, differentially expressed genes (DEGs), quantitative real-time polymerase chain reaction (qRT-PCR)

## Abstract

Tree peony (*Paeonia suffruticosa* Andrews) is a perennial woody shrub bearing large and colorful flowers. However, the flowering period is short and relatively uniform, which to an important extent hinders the cultivation and exploitation of ornamental peonies. In this study, the segregation of an F_1_ population derived from *P. ostti* ‘Feng Dan’ (an early-flowering cultivar) × *P. suffruticosa* ‘Xin Riyuejin’ (a late-flowering cultivar) was used to screen and analyze candidate genes associated with flowering period of the two parents. Extreme early- and late-flowering genotypes of the F_1_ population at full-bloom stage were sampled to establish an early-flowering mixed pool (T03), a late-flowering mixed pool (T04), a late-flowering male pool (T01), and an early-flowering female pool (T02), using the Sequencing By Synthesis (SBS) technology on the Illumina HiSeq TM2500 platform. A total of 56.51 Gb of clean reads data, comprising at least 87.62% of Quality30 (Q30), was generated, which was then combined into 173,960 transcripts (N50 = 1781) and 78,645 (N50 = 1282) unigenes, with a mean length of 1106.76 and 732.27 base pairs (bp), respectively. Altogether, 58,084 genes were annotated by comparison with public databases, based on an E-value parameter of less than 10^−5^ and 10^−10^ for BLAST and HMMER, respectively. In total, 291 unigene sequences were finally screened out by BSR-seq (bulked segregant RNA-seq) association analysis. To validate these unigenes, we finally confirmed seven unigenes that were related to early and late flowering, which were then verified by quantitative real-time PCR (qRT-PCR). This is the first reported study to screen genes associated with early and late flowering of tree peony by the BSA (bulked sample analysis) method of transcriptome sequencing, and to construct a high-quality transcriptome database. A set of candidate functional genes related to flowering time was successfully obtained, providing an important genetic resource for further studies of flowering in peony and the mechanism of regulation of flowering time in tree peony.

## 1. Introduction

Tree peony (*Paeonia suffruticosa* Andrews) is a deciduous shrub with large and colorful flowers that belongs to Sect. *Moutan* DC., genus *Paeonia*, family Paeoniaceae [1,2]. There are nine wild species in Sect. *Moutan* DC., all of which are unique to China [3]. Tree peonies therefore originated in China, and they have a long history of cultivation and use as ornamental plants. Over time, they have been introduced from China, either directly or indirectly, to many other countries throughout the world. Tree peonies possess abundant germplasm resources, and more than 1200 varieties may be recognized, following long-term natural and artificial selection [4,5]. Generally speaking, the individual flowers of any particular strain last for 10 days, but the flowering period from the first to the last flower is only 20 days, a very limited time period. Mid-season peonies predominate in the various different cultivation regions, with smaller numbers of early- and late-flowering varieties. Flowering characteristics and particular flowering period are key properties of the ornamental peony, and to an extent they have limited the commercial development of the plant [6]. Consequently, further studies of the molecular mechanisms that regulate flowering in peony are potentially of great significance for the manipulation and prolongation of the flowering period.

In recent years, with the rapid development and application of genetics and molecular biology, the functions of a large number of genes related to flowering have been extensively studied, especially in model plants with sequenced genomes. Results have shown that there are four pathways that regulate the mechanism of plant flowering at the molecular level. These comprise regulation by photoperiod, by vernalization, by gibberellins, and by an autonomous pathway; together, these control the timing of flowering in response to endogenous and environmental signals [7,8,9,10,11,12].

*Arabidopsis* possesses five phytochrome (PHY) genes (*PHYA–PHYE*) and two cryptochrome (CRY) genes (*CRY1* and *CRY2*) that are presumably involved in phase-setting under white-light-and-dark cycles [13]. These photoreceptors determine the activity of the flowering gene *CO*, which encodes a zinc-finger protein. CO acts as a floral activator and as a mediator of the circadian clock [14] to regulate the expression of *FT* (*FLOWERING LOCUS T*) and hence control flowering. *PHYA*, *CRY1*, and *CRY2* all promote flowering, whereas *PHYD* and *PHYE* inhibit flowering [15,16,17]. Vernalization causes suppression of expression of the central flowering repressor gene *FLC (FLOWERING LOCUS C)*; the suppression is stable for the remainder of the life of the plant, but expression returns to a high level in the following generation. Since the level of gene expression does not change during the entire process, the suppression of *FLC* by vernalization is not genetic [18]. In the unisexual flowers of cucumber, the plant hormone gibberellic acid (GA) largely determines flower development, especially the regulation of sepal/petal development in female flowers [19]. The autonomous flowering pathway accelerates flowering independently of day length by inhibiting the expression of *FLC* [20], and several genes are known to be involved, such as *LUMINIDEPENDENS* (*LD*), *FLOWERING LOCUS CA* (*FCA*), *FLOWERING LOCUS D* (*FLD*), *FLOWERING LOCUS Y* (*FY*), *FLOWERING LOCUS VE* (*FVE*), *FLOWERING LOCUS KH DOMAIN* (*FLK*), and *FLOWERING LOCUS PA* (*FPA*) [21,22,23,24,25]. The photoperiod and vernalization pathways are mainly regulated by environmental factors such as light and a low-temperature signal, respectively. On the other hand, the gibberellin and autonomous pathways are mainly regulated by developmental factors [26]. The transcriptional regulation of two genes determining flowering time, *SUPPRESSOR OF OVEREXPRESSION OF CONSTANS 1* (*SOC1*) and *FT* [27], and two floral meristem identity genes, *APETALA1* (*AP1*) and *LEAFY* (*LFY*) [28,29] has been shown to integrate these four pathways responsible for the regulation of flowering. These genes may, under particular circumstances, act either in isolation or in concert to activate downstream flower meristem genes that then initiate plant flowering.

However, regulation of flowering in woody perennials and in herbaceous species is very different. In perennial plants, flowering occurs following a transition between vegetative and reproductive growth that occurs at sexual maturity, after a juvenile period. Thus, Hsu et al. [30] reported that in woody perennial poplar (*Populus* spp.), *FLOWERING LOCUS T1* (*FT1*) and *FLOWERING LOCUS T2* (*FT2*) coordinate the repeated cycles of vegetative and reproductive growth, revealing that in response to winter temperatures, *FT1* determines reproductive onset, whereas *FT2* responds to warm temperatures and long days in the growing season and promotes vegetative growth and inhibition of bud set. Li et al. [31] explored the differences of leaf and peel color change between red and green walnut by transcriptome analysis and identified 3083 differentially expressed genes (DEGs) between red and green walnut peel at the ripening stage. Ma et al. [32] investigated the effects of low-temperature treatment on stamen petaloidy in rose (*Rosa hybrida*) and revealed that low temperatures increase petal number, at least to some extent. In tree peony, *PsTm6*, belonging to the MADS-box gene family, was found to influence stamen petaloidy and flower shape formation [33]. Zhang et al. [34] isolated *PsSOC1* from tree peony and determined its expression pattern during dormancy; furthermore, they investigated the regulatory mechanisms controlling flowering time in transgenic *Arabidopsis*. The results suggested that *PsSOC1* may be an important target for the genetic manipulation of dormancy release and flowering time in tree peony. Using transcriptome sequencing technology and by comparison with the non-repeat-flowering tree peony cultivar (× *P. suffruticosa* ‘Luo Yang Hong’ (LYH)), Zhou et al. [35,36] revealed eight DEGs that were potential candidates for determining repeat flowering in the repeat-flowering cultivar [*Paeonia* × *lemoinei* ‘High Noon’ (HN)]. Four genes, *PsFT*, *PsVIN3*, *PsCO*, and *PsGA20OX*, were identified that likely play important roles in the regulation of the repeat-flowering process in tree peony. Furthermore, these researchers isolated *PsFT*, a close homolog of *FT* found in the cultivars HN and LYH, and identified its potential role in the regulation of flowering time in tree peony.

Bulked sample analysis (BSA) is a powerful tool for the rapid identification of genetic determinants underlying phenotypic variation. It is applicable to both selected and pooled individuals, and it has been used extensively in gene mapping through bulked segregant analysis with biparental populations, in the mapping of molecular markers, such as single nucleotide polymorphisms (SNPs), and in pooled genome-wide association studies (GWAS), using extreme variants in two groups with contrasting phenotypes [37,38]. In maize, pools were constructed of mutants and wild-type individuals for comparison by RNA sequencing (RNA-Seq) and, using this approach, the *glossy 13* (*gl13*) gene [39], the *roothairless5* (*rth5*) gene [40], and the *Brown midrib2* (*bm2*) gene [41] were all successfully mapped and cloned. The three genes were related, respectively, to epicuticular waxes on the surfaces of seedling leaves, root hair initiation and elongation, and a reddish-brown coloration associated with reductions in lignin concentration and alterations in lignin composition. Ramirez-Gonzalez et al. [42] were able to identify putative SNPs across a major disease resistance gene for wheat yellow rust, the *Yr15* locus, using BSA combined with RNA-Seq in an F_2_ population to generate high-density genetic maps across target loci in polyploid wheat; they finally mapped *Yr15* to a 0.77-cM interval. In sunflower, in the absence of a reference genome, the putative locus *Pl_ARG_* conferring resistance to downy mildew was successfully verified by combining BSA with next-generation sequencing (NGS) and *de novo* assembly of the sunflower transcriptome, leading to SNP discovery through a sequence resource that combined reads that originated from two sunflower species [43].

For tree peony, resolution of the molecular mechanisms underlying the regulation of flowering, including time of flowering, is an important and complex problem and only limited progress has been made to date. In this study, we used bulked segregant RNA-seq (BSR-seq) technology for the first time to detect DEGs in 3 lines of early- and late-blooming flowers, selected from an F_1_ population derived from *P. ostti* ‘Feng Dan’ (an early-flowering cultivar) × *P. suffruticosa* ‘Xin Riyuejin’ (a late-flowering cultivar). We aimed to identify flowering-time-related candidate genes by comparing the transcriptomes of four different bulked pools of flowers, each selected at full-bloom stage: T01 (male bulk, ‘Xin Riyuejin’, late-flowering), T02 (female bulk, ‘Feng Dan’, early-flowering), T03 (20 early-blooming flowers), and T04 (20 late-blooming flowers). A set of candidate functional genes related to flowering time was successfully obtained, providing a rich genetic resource for further study of the molecular regulation of flowering initiation and timing in peony. In addition, the SSR and SNP molecular markers identified will be useful in the analysis of genetic evolution, genetic diversity, and population structure, and in genome-wide association studies (GWAS) of tree peony.

## 2. Results

### 2.1. Sequence Assembly and Annotation of Functional Genes

Four expression libraries of tree peony were sequenced, which generated 37,069,313 reads from T01, 33,883,337 reads from T02, 82,518,974 reads from T03, and 71,040,683 reads from T04, respectively. After data filtering, a total 56.51 Gb of clean data was obtained. The Q30 value was not less than 87.62%, and the GC content of each sample was between 40 mol % and 60 mol %. Furthermore, the ratio for T03 and T04 reached 85.60% and 85.18%, respectively (Table 1). A total of 173,960 transcripts and 78,645 unigenes were combined and assembled from scratch using Trinity software. The average length for a contig, a transcript, and a unigene was 59.211 bp, 1106.74 bp, and 732.27 bp, respectively. The N50 length for a transcript and a unigene was 1781 bp and 1282 bp, respectively (Table S1). The length distribution for all the combination unigenes is shown in Figure S1. A total of 58,084 genes were annotated by comparison with public databases; for the Nr database, the proportion annotated was 47.8%; for Swiss-Prot, it was 29.8%; for GO, 26.4%; for COG, 13.7%; for KOG, 27.4%; for KEGG, 16.5%; and for Pfam, 31.2%. The E-value parameter for BLAST and HMMER was <10^−5^ and <10^−10^, respectively.

### 2.2. Analysis of Differentially Expressed Genes (DEGs)

**Figure 1:**
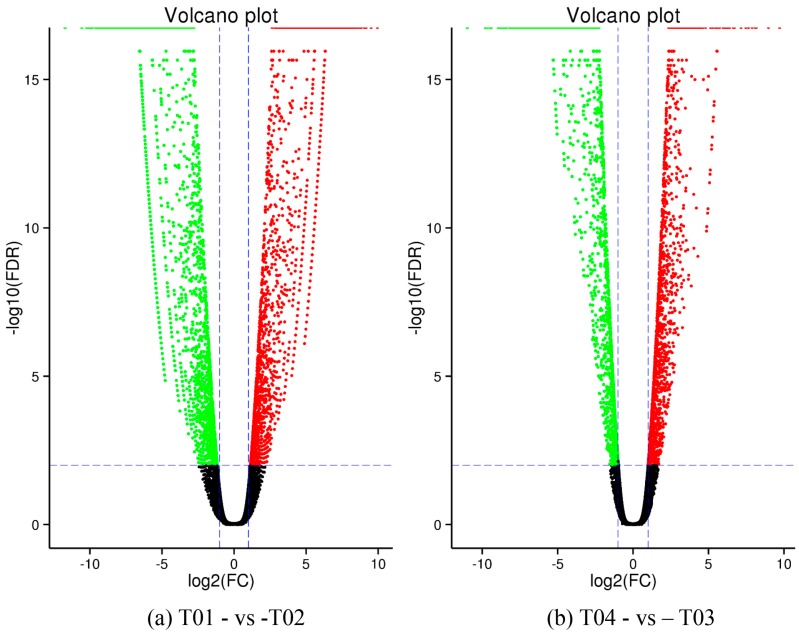
Volcano plot analysis of differentially expressed genes (DEGs) for the pairwise comparisons T01- vs. -T02 (**a**) and T04- vs. -T03 (**b**).

**Figure 2:**
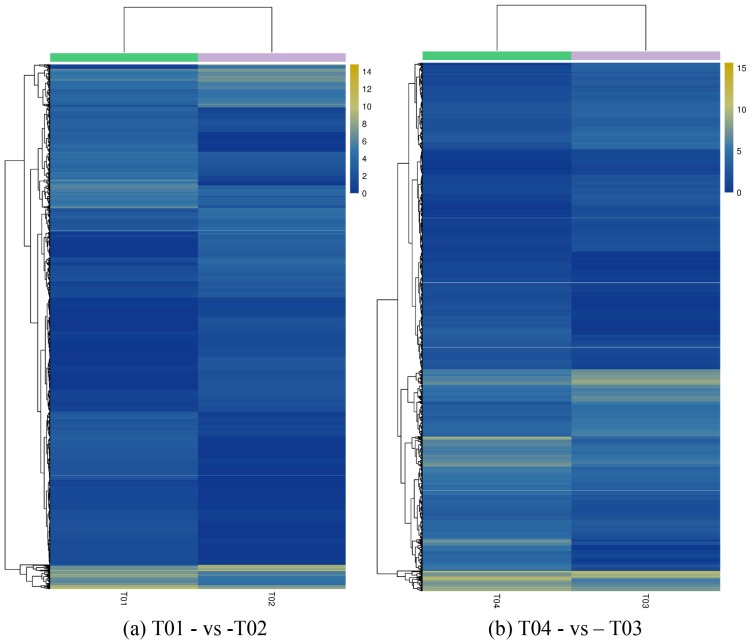
Hierarchical cluster analysis of differentially expressed genes (DEGs) for the pairwise comparisons T01- vs. -T02 (**a**) and T04- vs. -T03 (**b**). Note: Different columns represent different samples, different rows represent different genes, and different colors represent values of log_2_ (fragments per kilobase of transcript per million mapped reads (FPKM + 1)) to indicate different gene expression levels in the samples.

DEGs were identified by EBseq [44] using a False Discovery Rate (FDR) <0.01 and a Fold Change (FC) ≥2, where FC represents the ratio of expression between the two samples (groups). The total of 4789 DEGs that were identified from group T01- vs. -T02 was more than that from T04- vs. -T03; thus, for T01- vs -T02 there were 2309 up-regulated and 2480 down-regulated genes, whereas for T04- vs. -T03 there were 1879 up-regulated and 1094 down-regulated genes. Compared to T01 and T02, the total number of DEGs for T03 was more than 4000; analogously, compared to T01 and T02, the total number of DEGs for T04 was more than 3000 (Table 2). The volcano plot in Figure 1 shows that there were a large number of DEGs between T01 (late-flowering pool) and T02 (early-flowering pool), and between T04 (late-flowering pool) and T03 (early-flowering pool), and that the number of DEGs between the two parent groups was more than between the two mixed groups, whether up-regulated or down-regulated. DEGs with similar patterns of behavior were revealed through a hierarchical cluster analysis. Although the expression levels of DEGs were different between T01 and T02, as compared to between T03 and T04 (Figure 2), the differences between the two groups were similar, indicating that the DEGs identified from the two pairs of groups may be closely related to the mechanism of regulation of early and late flowering.

The DEGs were aligned to several public databases to obtain functional annotations (E-value ≤ 1 × 10^−5^). Consequently, among the six groups, almost all the unigenes were annotated to the Nr database; fewer were annotated to the Pfam and Swiss-Prot databases, and only a small proportion to the KEGG and COG databases (Table 3).

**Figure 3:**
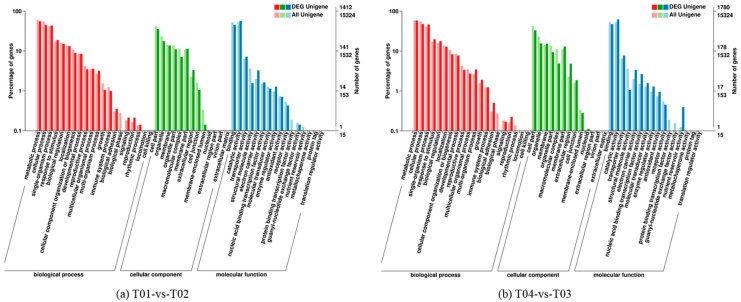
GO database annotation of differentially expressed genes (DEGs) for the pairwise comparisons T01- vs. -T02 (**a**) and T04- vs. -T03 (**b**).

The GO (Gene Ontology) database is an internationally standardized database of gene functional classification. Overall, for the two pairwise comparisons T01- vs. -T02 and T04- vs. -T03, 1412 and 1780 DEG unigenes, respectively, were classified into 13 “cellular component”, 16 “molecular function”, and 20 “biological process” categories. With regard to cellular component, “organelle” (17.56% for T01- vs. -T02, 15.56% for T04- vs. -T03) and “cell part” (35.62%, 32.98%) were the most prevalent categories. For molecular function, the most prevalent were “transporter activity” (7.22%, 7.75%), “binding” (44.83%, 47.25%), and “catalytic activity” (57.08%, 62.36%); and for biological process, they were “cellular processes” (44.62%, 47.81%) and “metabolic processes” (55.52%, 58.26%). The GO annotations for the two pairwise comparisons are shown in Figure 3.

**Figure 4:**
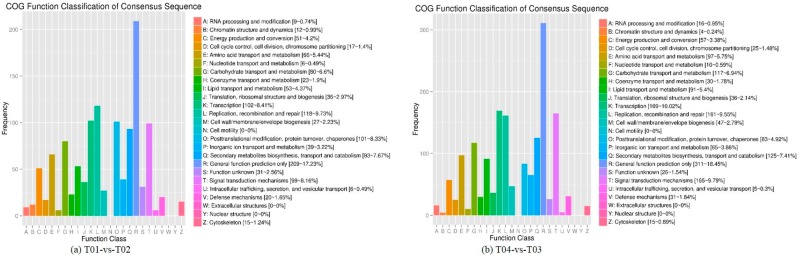
COG classification of differentially expressed genes (DEGs) for the pairwise comparisons T01- vs. -T02 (a) and T04- vs. -T03 (**b**). Note: The *x*-axis is the COG category; the *y*-axis is the number of genes.

The COG (Clusters of Orthologous Groups (of proteins)) database is based on phylogenetic relationships of gene products across bacteria, algae, and eukaryotes. Following the GO classification of DEGs described above, categorization by COG revealed enrichment in 22 categories, of which the largest proportion (209 or 17.23% for T01- vs. -T02, 311 or 18.45% for T04- vs. -T03) were assigned to the “general function” prediction category. There were no DEGs represented in the categories “extracellular structures,” “cell motility,” and “nuclear structures.” Otherwise, the least-represented categories were “intracellular trafficking”, “secretion”, and “vesicular transport,” with six DEG unigenes (0.49%) in the T01- vs. -T02 group, and “chromatin structure and dynamics,” having four unigenes (0.24%) in the T04- vs. -T03 group (Figure 4).

**Figure 5:**
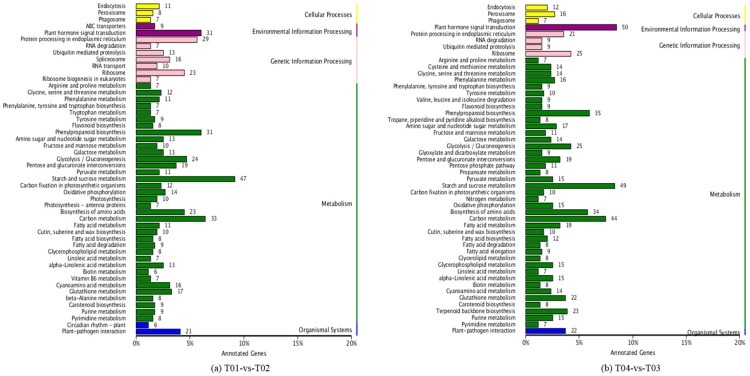
KEGG pathway classification of differentially expressed genes (DEGs) for the pairwise comparisons T01- vs. -T02 and T04- vs. -T03. Note: (**a**) The *x*-axis shows the number of annotated genes and the proportion of DEGs corresponding to each pathway category and the *y*-axis shows the 50 out of the 118 pathway categories that contain more than six DEGs; (**b**) As for (**a**), except that the *y*-axis shows the 50 out of 113 pathway that contain more than six DEGs.

The KEGG (Kyoto Encyclopedia of Genes and Genomes) database enables the systematic analysis of genes in relation to a range of biological functions, in principle from the molecular to the population level. A KEGG annotation of the DEGs of the T01- vs. -T02 pairwise comparison revealed that 512 DEGs were assigned to five top-level categories, including “cellular processes”, “environmental information processing”, “genetic information processing”, “metabolism”, and “organismal systems”. These DEGs were in turn mapped onto 118 pathway categories, of which the top three were “starch and sucrose metabolism” (47, 9.18%), “carbon metabolism” (33, 6.45%), “plant hormone signal transduction” (31, 6.05%), and “phenylpropanoid biosynthesis” (31, 6.05%) (Figure 5a). The T04- vs. -T03 pairwise comparison produced broadly similar results to the T01- vs. -T02 comparison, with 588 DEGs assigned to the same five top-level categories and then mapped on to 113 pathway categories; thus, 50 (8.50%), 49 (8.33%), and 44 (7.48%) of these DEGs mapped onto “plant hormone signal transduction”, “starch and sucrose metabolism”, and “carbon metabolism”, respectively (Figure 5). Taken together, the two sets of data clearly indicated the importance of metabolic changes in flower development and differentiation.

### 2.3. Verification of Candidate Genes through Quantitative Real-Time PCR (qRT-PCR)

**Figure 6:**
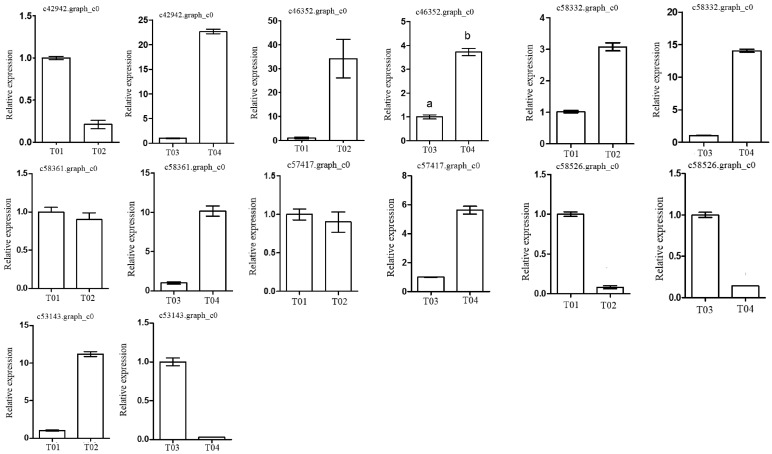
Validation by qRT-PCR of seven differentially expressed unigenes (DEGs) isolated from the four different pools (T01–T04) of flower petals of tree peony. Data from qRT-PCR were normalized relative to the Actin gene of *P. suffruticosa* (F: GGTCTATTCTTGCTTCCCTCAG; R: GAACTCACTATCAAACCCTCCAG). The *x*-axis denotes the four pools, T01, T02, T03, and T04, representing the late-flowering male pool, the early-flowering female pool, the early-flowering mixed pool, and the late-flowering mixed pool, respectively. The *y*-axis denotes relative levels of gene expression and the values are expressed as the means of three replicates ±SD.

From the pairwise comparisons, a total of 291 DEGs that showed a significantly enriched association with known genetic loci were selected (based on a False Discovery Rate (FDR) < 0.01) (Table S2). These candidate genes were annotated using public databases including GO (Figure S2) and KEGG (Figure S3). On the basis of this annotation analysis, the seven DEGs that displayed the highest number of SNPs and associated loci and that had a greater likelihood of being associated with phenotypic traits were selected (Table 4), and the relevant information is shown in Table S3 and Appendix 1 (see Supplementary Materials 1). Next, to corroborate the RNA-Seq results and to investigate the dynamics of expression of DEGs in tree peony flowers, these seven key DEGs, together with one reference gene showing identical expression, were selected for qRT-PCR analysis. Figure 6 shows the differential expression levels for these seven key DEGs for the pairwise comparisons T01- vs. -T02 and T04- vs. -T03. The unigenes *c42942.graph_c0*, *c58332.graph_c0*, *c58361.graph_c0*, and *c57417.graph_c0* were expressed at a much higher level in T04 than in T03, yet they were expressed at comparable levels in the two parents. In contrast, *c46352.graph_c0* and *c53143.graph_c0* were expressed at a much higher level in T02 than in T01, but were expressed at comparable levels in both F_1_ pools. The unigene *c58526.graph_c0* was expressed at comparable levels in both the parents and the F_1_ pools. Additionally, in T02- vs. -T01, the expression levels of four DEGs associated with “carbohydrate transport and metabolism”, “hothead-like”, “reduced wall acetylation 4-like”, and “PTI 1-like tyrosine-protein kinase” were all observed to be down-regulated, whereas the expression levels of three DEGs associated with “plant invertase/pectin methylesterase inhibitor”, “K^+^ transporter”, and “peptidyl-prolyl *cis-trans* isomerase activity” were up-regulated. On the other hand, five DEGs related to “carbohydrate transport and metabolism”, “plant invertase/pectin methylesterase inhibitor”, “K^+^ transporter”, “hothead-like”, and “reduced wall acetylation 4-like” were down-regulated in T01- vs. -T02. In the second pairwise comparison, T04- vs. -T03, two DEGs identified as “PTI 1-like tyrosine-protein kinase” and “peptidyl-prolyl *cis-trans* isomerase activity” were up-regulated. The sequencing of the seven key DEGs are listed in Appendix 1. The results obtained from the qRT-PCR analysis were completely consistent with those obtained by RNA-Seq, thereby demonstrating the reliability of the RNA-Seq data.

### 2.4. Simple Sequence Repeats (SSRs) and Single Nucleotide Polymorphisms (SNPs)

Structural analysis of unigenes (length > 1 kb) identified 6678 SSRs, which contained six types of SSR, as follows: mono-nucleotide (4347; 65.09%), di-nucleotide (1443; 21.61%), tri-nucleotide (833; 12.47%), tetranucleotide (35; 0.52%), penta-nucleotide (8; 0.12%), and hexanucleotide repeat (12; 0.18%) (Table 5). Furthermore, we also detected 219,291 SNPs, applying the following stringent screening criteria: (1) the continuous single-base mismatch was not more than 3, within a range of 35 bp; (2) SNP quality values were greater than 2, based on a sequence-depth normalization. SNPs could be classified as homozygous SNPs (with only one allele) and heterozygous SNPs (Heterozygosity: two or more digits) according to the number of alleles (Allele) of the SNPs, i.e., the number of different bases supported by sequencing reads. Table 6 shows the statistical results of SNP loci.

## 3. Discussion

Plant flowering results from a transition from vegetative growth to reproductive growth, and time of flowering is regulated by a series of gene–environment interactions. The molecular mechanism of flowering plays a crucial role in plant growth and development, and it has become a “hot topic” in plant science. A full understanding is, self-evidently, of great importance to the identification of genes involved in plant developmental regulation. To date, a large number of genes have been discovered based on the increasing amount of sequence data available and gene expression patterns in plant organs such as flowers, leaves, and fruits. Because transcriptome sequencing can be undertaken irrespective of whether the species of interest has a sequenced reference genome, transcriptome sequencing has been recognized to be the most effective way of mining functional genes.

In the present study, the BSR-Seq method was used, employing the Illumina HiSeq TM2500 platform to screen and identify genes involved in time of flowering in tree peony. Petals from early- and late-flowering samples of an F_1_ population derived from a cross between two cultivars with flowering times (*P. ostti* ‘Feng Dan’ (early flowering) × *P. suffruticosa* ‘Xin Riyuejin’ (late-flowering)) were used for transcriptome sequencing, and a large number of gene sequences were obtained within a short period of time. Thus, a total of 56.51 Gb of clean data was acquired by transcriptome sequencing of samples from four groups, viz. T01 (male bulk, ‘Xin Riyuejin’, late-flowering), T02 (female bulk, ‘Feng Dan’, early-flowering), T03 (20 early-blooming flowers), and T04 (20 late-blooming flowers). Ultimately, a total of 78,645 unigenes were identified by de novo assembly.

There has been rapid progress recently in the application of transcriptome sequencing to peony, with a range of different tissues having been selected for different purposes. For example, the different developmental stages of peony flower buds were used for transcriptome sequencing by Shu et al. [45], who constructed the first cDNA library of peony and obtained 2241 expressed sequence tags (ESTs). Gai et al. [46] used the 454 GS FLX platform to transcriptome-sequence the dormant buds of ‘Feng Dan’ and revealed the molecular mechanism of dormancy in tree peony. A total of 50,829 unigene sequences with an average length of 585 bp were obtained from the petals of ‘Luoyang Hong’ using the HiSeq TM2000 platform, and from this the mechanism of anthocyanin synthesis in peony cut flowers was clarified [47]. Li et al. [48] carried out transcriptome analysis of peony seeds at different developmental stages and identified 175,875 contigs; subsequently, 2182 differentially expressed unigenes were screened and a large number of DEGs involved in fatty acid metabolism were identified, providing a molecular basis for potential strategies to increase the yield of peony seed oil. In comparison with these results, it is gratifying that the number of unigenes in the present study was as high as 78,645, which is 35 times the number of EST sequences of the flower bud transcriptome, and more than the numbers reported by Zhang and Li et al. [47,48]. Therefore, the present study provides a comprehensive and high-quality genetic resource for research on peony and its mechanism of flowering.

Four peony transcriptome samples (T01–T04) were sequenced in this study and, following de novo assembly and alignment to publicly available databases, a total of 28,347 annotated unigenes, 36.04% of the total, were identified. The GO classification of T01- vs. -T02 was compared with that of T03 vs. T04, and both were found to be enriched in 49 functional categories, mainly “response to stimulus”, “biological regulation”, “cell part, “organelle”, “catalytic activity”, and “binding”. Subsequently, KEGG pathway analysis revealed that the principal enriched pathways comprised “plant hormone signal transduction”, “metabolic pathways”, and “secondary metabolic pathways”. The above analysis therefore indicated that genes related to plant hormone signaling, cell metabolism, and secondary metabolism play important roles in flower development in peony. In addition, comparing T04 (late-flowering mixed pool) vs. T01 (late-flowering pool), there were 3257 DEGs, of which 1398 were up-regulated and 1859 were down-regulated; comparing T03 (early-flowering mixed pool) vs. T02 (early-flowering pool), there were 4415 DEGs, of which 1398 were up-regulated and 1859 were down-regulated.

Following the BSR-seq association study, a total of seven genes involved in peony flowering were selected for functional annotation, notably *c57417.graph_c0*, encoding a “removing wall acetylation” (RWA) protein, an epigenetic gene. This gene plays an important role in acetylation processes in the plant cell, and has a direct impact on the formation of cell wall polysaccharides and on related cellular functions; it affects morphology, flowering, and other plant traits, depressing the expression of *FLC* and initiating flowering [24]. Interestingly, the expression level of the gene in the late-flowering pool was lower than in the early-flowering pool, indicating that the action of the gene might be unique. The gene encoding a K^+^-ion transporter protein (*c58332.graph_c0*) relates to one of the three principal mineral elements that are essential for plant growth and changes in its expression could therefore have important physiological and biochemical effects. The gene *c58526.graph_c0* could regulate plant flowering via a signal transduction process.

These screened genes were verified via qRT-PCR, which showed that although the expression levels of the genes in the two pairwise comparisons (T01- vs. -T02 and T04- vs. -T03) were different, the trend was consistent. The quality of the peony petal transcriptome database constructed in this study was high, and the database provides an accurate and information-rich resource for future research related to peony flowering. However, because of the large apparent variation in flowering time phenotype in the F_1_ generation, which may reflect the large genetic variation between the two parental cultivars, *P. ostti* ‘Feng Dan’ (early-flowering) and *P. suffruticosa* ‘Xin Riyuejin’ (late-flowering), and heterosis and inbreeding depression may occur in the F_1_ generation. These factors could cause epistasis or genetic interaction, and this could affect the functioning of the genes identified [49,50]. All these functionally annotated genes should therefore be further validated in future studies. 

In addition to the seven genes mentioned above, other genes related to the four genes *PsFT*, *PsVIN3*, *PsCO*, and *PsGA20OX* are known to play important roles in the regulation of the repeat-flowering process in tree peony [37,38], including the unigenes *c23725.graph_c0* and *c43875.graph_c0*, which may be related to the MADS-box protein SOC1, *c6585.graph_c0*, which may be related to gibberellin 20 oxidase, and the *c51656.graph_c0*, *c30761.graph_c0*, and *c30761.graph_c0* unigenes, which may be related to the VIN3 protein. Further attention needs to be paid to these particular unigenes in future work.

In this study, the identification of genes potentially associated with peony flowering has shed light on potential control mechanisms and their possible commercial application in peony. Further studies are needed to elucidate specific functions and possible interactions at the molecular level. In other respects, the SSR and SNP markers that have been identified will be useful for characterizing the genetic diversity of peony genetic germplasm resources and for genome-wide association studies (GWAS), thus providing a theoretical basis for the conservation of germplasm and for the molecular-assisted breeding of peony.

## 4. Materials and Methods

### 4.1. Plant Materials

*P. ostti* ‘Feng Dan’ (an early-flowering cultivar) × *P. suffruticosa* ‘Xin Riyuejin’ (a late-flowering cultivar) were hybridized, and the two parents and 20 early- and late-flowering individuals from the F1 population selected at the full-bloom stage were used to construct BSA segregation groups [51]. The flowering times of the F1 population and of the parent cultivars are given in Table S4 (Supplementary Materials 2). Flower petals from the parent cultivars and the 40 F1-population individuals were collected from a farm at the Henan University of Science and Technology Experimental Station, Luoyang, China (34°60 N, 112°42 E) in April 2015. All samples were frozen in liquid nitrogen immediately after collection in the field and were stored in a −80 °C freezer pending RNA extraction.

### 4.2. RNA Extraction and Illumina Sequencing

Total RNA was extracted from tree peony petals using a RNA prep Pure Plant Kit (Polysaccharides & Polyphenolics-rich) (Tiangen, Beijing, China). RNA quality and concentration were assessed by electrophoresis on a 1.2% agarose gel and using a NanoDrop 2000 UV-Vis Spectrophotometer (Thermo Fisher Scientific, Wilmington, DE, USA), respectively. RNA samples were taken from each of four groups: T01 (male bulk, ‘Xin Riyuejin’, late-flowering), T02 (female bulk, ‘Feng Dan’, early-flowering), T03 (20 early-blooming flowers), and T04 (20 late-blooming flowers). These were then analyzed by Biomarker Technologies Corporation (Beijing, China).

The bulked RNA was enriched for mRNA using Oligo (dT) Beads and then randomly-cleaved into short fragments. First-strand cDNA was synthesized from mRNA using random-hexamer primers. DNA polymerase I, RNase H, dNTPs, and buffer were used to synthesize the second-strand cDNA. The double-stranded cDNA was then purified using an AMPure XP beads kit and end-repaired, and then a single nucleotide A (adenine) addition was ligated to the sequencing adapters. The required fragments were selected using AMPure XP beads and enriched by PCR amplification to create the final cDNA library. Finally, the mRNA-seq library was constructed for paired-end sequencing (reads = 125 bp) on the Illumina HiSeq TM2500 sequencing platform (Biomarker Technologies Corporation Beijing, China). In addition, library concentration and insert size were assessed using Qubit 2.0 Fluorometer (Life Technologies, Carlsbad, CA, USA) and an Agilent 2100 Bioanalyzer (Agilent Technologies, Waldbronn, Germany), and Q-PCR (an Applied Biosystems Step One machine, Applied Biosystems, Foster City, CA, USA) was used to accurately quantify the effective concentration of the library and to ensure its quality.

### 4.3. De Novo Assembly and Quality Control

In order to obtain high-quality clean reads data for de novo assembly, the raw data reads were filtered to remove adaptor sequences and low-quality sequences containing unknown bases (reads with ‘N’ bases) > 10% and with a Q-value < 20. At the same time, the Q20, Q30, and GC content of the clean data were calculated. All the downstream analyses were based on high-quality clean data. After obtaining the clean data, reads assembly was accomplished using Trinity software (http://trinityrnaseq.sourceforge.net/) [52]; the diversiform clean reads were assembled with transcripts characterized by the same subcomponent being regarded as a gene and the longest transcript of each gene being selected and defined as the unigene.

### 4.4. Unigene Functional Annotation and Gene Structure Analysis

No genomic data is available for peony. The functional annotation of unigenes was achieved using BLAST software [53] to search for similarity in public databases, and then the functions of unknown genes were inferred from the homology of annotated genes in the databases. We searched against the following public databases: Nr database (NCBI non-redundant protein sequences) (ftp://ftp.ncbi.nih.gov/blast/db/) [54], Swiss-Prot protein database, (http://www.uniprot.org/) [55], GO (Gene Ontology) (http://www.geneontology.org/) [56], COG (Clusters of Orthologous Groups) (http://www.ncbi.nlm.nih.gov/COG/) [57], KOG (euKaryotic Orthologous Groups) (http://www.ncbi.nlm.nih.gov/COG/) [58], and KEGG (Kyoto Encyclopedia of Genes and Genomes) (http://www.genome.jp/kegg/) [59]. After prediction of the translated amino acid sequence of the unigene, annotation of the amino acid sequence was obtained by aligning HMMER [60] with the Pfam Protein family database (http://pfam.xfam.org/) [61].

The prediction of the unigene coding region sequence and its corresponding amino acid sequence was realized via TransDecoder software (http://sourceforge.net/projects/transdecoder/). In addition, the MISA (MIcroSAtellite identification tool) software (http://pgrc.ipk-gatersleben.de/misa/misa.html) was used to analyze unigene sequences.

### 4.5. Gene Expression Quantification

Reads of each sample sequenced were aligned with the unigene library using Bowtie [62], and then the level of expression was estimated based on the alignment results and RSEM [63]. Subsequently, the expression level of the unigene was expressed as FPKM (Fragments Per Kilobase of transcript per Million mapped reads) [64]. FPKM can eliminate the influence of the difference between gene length and the amount of sequencing on the calculation of gene expression, hence permitting gene expression differences to be compared among different samples.

### 4.6. Analysis of Genes with Differential Expression (DEGs)

The recognized effective Benjamini–Hochberg method was used to correct the significant *p*-value that was obtained from the original hypothesis test among the differentially expressed genes (DEGs) analysis. Finally, the corrected *p*-value, the False Discovery Rate (FDR), was used as a key indicator of DEGs screening and a false-positive test was performed to reduce the expression value of a large number of genes independently. The DEGs (FDR < 0.01 and Fold Change (FC) ≥ 2) were identified by EBseq [44], of which FC represents the ratio of expression between two samples (groups). In our study, four groups (T01, T02, T03, and T04) were compared with each other (T01- vs. -T02, T03 vs. T01, T03 vs. T02, T04 vs. T01, T04 vs. T02, and T04- vs. -T03) to screen out genes related to early and late flowering of peony. A volcano plot was created to intuitively show the significance of the DEGs, and a MA diagram was created to identify the distribution of gene expression abundance and differential multiples between pairs of groups. Furthermore, a hierarchical cluster analysis that clustered genes with the same or similar expression was performed to display the differential expression patterns of genes under different experimental conditions. Finally, the identified DEGs were subjected to functional annotation by databases including GO, COG, and KEGG.

### 4.7. BSR-Association Study and Candidate Genes Identification

The reads and unigene sequences were compared for each sample using STAR (http://code.google.com/p/rna-star/) [65] and the Single Nucleotide Polymorphism (SNP) site and then identified by GATK (https://www.broadinstitute.org/gatk/) [66]. In order to ensure the accuracy of subsequent analysis, loci for which the read support was <3 were first filtered out. To obtain high-quality reliable SNP sites and to identify through association analysis loci differing between T03 and T04, SNP discrepant-type loci were filtered out through T03 + T01 and T04 + T02, homoplastically, and then SNP consistent-type loci were filtered out through T03 + T04. The Euclidean Distance (ED) algorithm was used to calculate the region that related to the objective gene linkage. The arithmetic was based on the depth of the SNP discrepancy between T03 and T04 and the ED value was calculated according to the following formula (Equation (1)):

(1) $$\mathrm{ED}=A\sqrt{{\left(A_{T03}-A_{T04}\right)}^{2}+{\left(C_{T03}-C_{T04}\right)}^{2}+{\left(G_{T03}-G_{T04}\right)}^{2}+{\left(T_{T03}-T_{T04}\right)}^{2}}$$

The higher the ED value, the greater the difference between T03 and T04 in SNPs. In order to eliminate the difference in the ED results caused by differences in sequencing between the two mixed pools, we used the frequency of each base at each locus instead of the absolute value to calculate the ED value, and this was raised to a power of 5 (ED5) to eliminate the noise generated by small variations in the estimations in our study.

Because of the lack of genomic information for peony at the chromosome level, we used the following analysis strategy in order to determine a credible association area: (1) The ED values for all loci were calculated and ED = 0.74 was used as the associated threshold; (2) Loci that exceeded the association threshold were selected and served as candidate association loci; (3) Statistics of the number of SNPs and candidate loci for every unigene difference between T03 and T04 were recorded; (4) The probability of the accumulation of association sites in each unigene was calculated from the hypergeometric distribution, calculated as follows (Equation (2)):

(2) $$P=1-\overset{y-1}{\underset{x=0}{\sum }}\frac{\left(\begin{array}{c} K\\ x \end{array}\right)\left(\begin{array}{c} M-K\\ N-x \end{array}\right)}{\left(\begin{array}{c} M\\ N \end{array}\right)}$$

In the above formula, M represents the total number of differences in SNPs between the T03 and T04 mixed pools, K represents the total number of all candidate association loci, and Y represents the number of candidate association loci in the unigenes; (5) The Benjamini–Hochber method was used to multiply and correct the test for the probability of each unigene enrichment-associated locus, and then calculate the FDR value; (6) Unigenes with significant enrichment-associated sites (FDR < 0.01) were screened. Thereafter, the identified SNP-associated genes were subjected to functional annotation by databases including GO, COG, and KEGG.

### 4.8. Quantitative Real-Time PCR (qRT-PCR) Verification of Candidate Genes

To study candidate gene expression profiles in the four samples (T01, T02, T03, and T04), we selected the relatively stably expressed peony *Actin* gene as a reference gene for qRT-PCR [67]. cDNA synthesis was performed as described earlier (Section 4.4). Quantitative real-time PCR was performed on a CFX ConnectTM Real-Time PCR System (Bio-Rad, Hercules, CA, USA). The primer information for *Actin* and the candidate genes is shown in Table A3. Each PCR reaction was repeated three times and the volume of the qPCR reaction was 20 μL. The cycling protocol consisted of 3 min at 94 °C, followed by 40 cycles of 15 s at 94 °C for denaturation, 15 s at 55 °C for annealing, and 20 s at 72 °C for extension. The specificity of the PCR reaction was assessed by the presence of a single peak in the dissociation curve after the amplification. Relative expressions of target genes were analyzed using the 2^−ΔΔCt^ algorithm [68], in which CT values of reference genes are calculated with a geometrical.

## 5. Conclusions

Flowering period is an extremely important parameter in the cultivation and commercial production of peonies as ornamental subjects. Our study is the first to screen the genes of early- and late-flowering in tree peony by the BSA analysis method of transcriptome sequencing and to construct a high-quality transcriptome database. A set of candidate functional genes related to flowering time was successfully obtained, providing a rich genetic resource for studies of peony flowering and establishing a foundation for more detailed studies of flowering-period regulation in tree peony. The development of SSRs and SNPs as molecular markers will be useful in the analysis of gene evolution, genetic diversity, and population structure, and for genome-wide association studies (GWAS) of tree peony. The data will also greatly assist breeding programs, and the conservation of germplasm in tree peony.

## Figures and Tables

**Table 1:** Results statistics and comparison of peony petal transcriptome sequencing and assembly.

Samples	Samples-ID	Base Number	Read Number	GC Content (%)	%≥Q30	Clean Reads	Mapped Reads	Mapped Ratio
male parent (♂)	T-01	9,332,641,596	37,069,313	44.69	87.82%	37,069,313	31,527,468	85.05%
female parent (♀)	T-02	8,531,057,760	33,883,337	44.82	87.62%	33,883,337	28,497,709	84.11%
Early flowering pool	T-03	20,778,247,714	82,518,947	44.61	88.16%	82,518,947	70,633,854	85.60%
Late flowering pool	T-04	17,887,013,878	71,040,683	44.68	88.02%	71,040,683	60,509,445	85.18%

Note: Base number: the total number of bases in Clean Data; Read Number: the total number of paired-end reads in Clean Data; GC Content: the GC content of Clean Data (G and C bases as a percentage of the total bases in Clean Data); %≥Q30: the percentage of bases in Clean Data for which the Quality Score is ≥30; Clean Reads & Mapped Reads: the number of clean reads and mapped reads (calculated as paired-ended); Mapped Ratio: the percentage of mapped reads in clean reads.

**Table 2:** The number of differentially expressed genes was calculated in four samples.

DEG Set	All DEG	Up-Regulated	Down-Regulated
T01- vs. -T02	4789	2309	2480
T03- vs. -T01	4211	1983	2228
T03- vs. -T02	4415	1932	2483
T04- vs. -T01	3257	1398	1859
T04- vs. -T02	3644	1484	2160
T04- vs. -T03	3783	1879	1094

Note: T01, male pool; T02, female pool; T03, the earliest-flowering individuals from the F1 population sampled at full-bloom stage to establish an early-flowering mixed pool; T04, the latest-flowering individuals from the F1 population sampled at full-bloom stage to establish a late-flowering mixed pool.

**Table 3:** The number of differentially expressed genes (DEGs) calculated for the four samples T01–T04.

DEG Set	Annotated	COG	GO	KEGG	KOG	Pfam	Swiss-Prot	NR
T01- vs. -T02	2606	815	1412	843	1321	1954	1816	2560
T03- vs. -T01	2911	978	1663	920	1424	2325	2118	2872
T03- vs. -T02	3004	1035	1754	960	1504	2450	2245	2972
T04- vs. -T01	2140	687	1183	714	1070	1679	1517	2104
T04- vs. -T02	2172	710	1198	744	1135	1703	1553	2138
T04- vs. -T03	2993	1063	1780	963	1478	2507	2294	2972

**Table 4:** DEGs displaying the highest number of SNPs and associated loci.

Unigene	Annotation	All Count	Asso Count	*p*-Value	FDR	Regulated	
						T01- vs. -T02	T04- vs. -T03
*c42942.graph_c0*	Carbohydrate transport and metabolism	4	4	0	0	down	down
*c46352.graph_c0*	Plant invertase/pectin methylesterase inhibitor	4	4	0	0	up	down
*c58332.graph_c0*	K^+^ potassium transporter	9	7	0	0	up	down
*c58361.graph_c0*	Hothead-like	13	12	0	0	down	down
*c57417.graph_c0*	Reduced wall acetylation 4-like	16	5	1.23 × 10^−9^	1.41 × 10^−6^	down	down
*c58526.graph_c0*	PTI 1-like tyrosine-protein kinase	14	4	4.19 × 10^−8^	2.10 × 10^−5^	down	up
*c53143.graph_c0*	peptidyl-prolyl *cis*-*trans* isomerase activity	8	3	2.05 × 10^−7^	9.16 × 10^−5^	up	up

**Table 5:** Types of simple sequence repeats (SSRs) identified in the transcriptome sequencing of tree peony.

Searching Item	Number
Total number of sequences examined	16,885
Total size of examined sequences (bp)	33,407,765
Total number of identified SSRs	6678
Number of SSR containing sequences	5191
Number of sequences containing more than 1 SSR	1170
Number of SSRs present in compound formation	333
Mono nucleotide	4347
Di nucleotide	1443
Tri nucleotid	833
Tetra nucleotide	35
Penta nucleotide	8
Hexa nucleotide	12

**Table 6:** SNP statistics for the transcriptome sequencing of the four tree peony pools (T01–T04).

Samples	Homozygosity	Heterozygosity	SNP Number
T01	109,252	81,140	190,392
T02	142,760	43,169	185,929
T03	40,888	166,550	207,438
T04	50,735	155,675	206,410

## Supplementary Materials

Supplementary materials are available online.
