# Supplementary material for: Screening of Genes Related to Early and Late Flowering in Tree Peony Based on Bulked Segregant RNA Sequencing and Verification by Quantitative Real-Time PCR
Source: Molecules. 2018 Mar 19;23(3):689. doi: 10.3390/molecules23030689 (PMC6017042; doi:10.3390/molecules23030689)
Supplement: Supplementary file 1 [file molecules-23-00689-s001.zip › Supplementary Materials/1. Supplementary Materials/Supplementary Materials 1.pdf]

Appendix 1　7 sequences related to flowering genes

>c42942.graph_c0
AGCCGATTTCTTAGTCCCAGCCATAGTTGAGTGACAAGCAACAACCATTAT
ATAAACCAAAATCAAGACAACATTATCTAATCAAATTTTCAAGATCTAAGCT
GCTTTCTTCAATGGCTTCCAGACCACTTCGTTACTTCCTCTATCTATACCTGT
ACCTCCTTTTTCTTCTCCAACTCCACAACTCATCCGCACAAGATGTTGTCAA
GGCTGCTTACTGGTTTCCAGGCAGTGAATTCCCAGTTTCCGACATCGACTC
CACTCTTTTTACTCATCTCATCTGTGCATTTGCCAACTTAGATACCCAAACC
AACCAACTTGTTATTTCATCTGCAAATACACCTTCTTTCTCCAGCTTTACAA
AAACCGTCCAACTGAAAAACCCTTCAGTTAAAACCCTCTTATCCATCGGAG
GGGGAAATGTGGCCTCGGAAACCTTCGCTTCAATGGCTAGTCAATCTAGTT
CACGTAAAACTTTTATTGATTCTTCGATCAAGTTGGCGAGAAGTTATGACTT
TCATGGCCTGGACTTGGATTGGGAGCACCAAACAATGAATTCTGAGATGAC
TTCCTTGGGTTTGCTCCTTGACGAGTGGAGAGCGGCTGTCGACACCGAGA
CCTCTGATTCTGGACTACCAAAGTTGATTCTGACAGCAGCAGTTCCATTCA
CGCCGCTGATTAATTCAATTGTTTATCCCACTGCATCTTTATCAAGGAACTTG
GACTGGATCAATGTAATGGCATATGACTTCTATGCACCAAACAGAGCTAGTG
CCTCAGGTGCAACACACTCGCATGCTGCTTTATACGATTCATCAAGTGATGT
TAGTGCAAGTTATGGGATTGAAAAATGGATTGAAAGGGGGGTTTCTTCAAA
GAAGATAGTAATTGGCTTTCCCTTCTACGGCTATGCATGGCTTCTTACAAGC
CCTAATAACCATGGCATTTTAGCACCTGCTAATGGACCAGATCTTTCGGTTG
GTGGATACGTCGGAAGTGATGGGTCCATTGGGTATAACCAGATCAAGGTGT
TTATAGGGCAAAAGGGTGCCACAACAGTGTTCAATGATACAATTGTTGCGG
ATTATTGCTACGTTGGGACGTCTTGGATTGGTTATGATGATGTGCAGACTATT
TCCACTAAGATTTCGTATGCTAAAGGAAAAAGGTTGCTTGGTTATTTTGCAT
GGCACGTTGGGTCTGATGATAGCTGGGTCCTTTCTAAACAAGCTTCACAAA
CATGGGGAGCTTAAATATTTGTTTCTGGTAACAGGACTAATAAACCATAATA
AAATAATGAACTATTTGATCAATGATAAATAAATATTAATATGGGCCTTACAA
GACCATTTTGTAACTGCTGCTTAAAAAACAATTTTGTTCTCTTCCTTTTGCA
TTTCTAAACTGAATTGTGATGGGTAGGAATGAAGTAAGTGTGTGATAAAATT
TCCATATCCAAGTATGTTCTTGTAGCCAATAAGATACACCTCATTATAAGATG
AACATATGATTAAGAACTCCATGACTCTACCTTAAGTTCATATGGCAAAACA
AACTCACAGGAATAACTTAATAATCAGATGATGTTTCGTTTACAACCAATAA
ACCACAGAGACAGTGAACTATGAACAAGTTAAACATATTATTATTTGTTTAT
AATCAACATTTCCTCACTTTTATTACTTTAATATTATGCCACTTTGTAATCACC
AAACAAGTGAAAATTACATTATACGAA

>c46352.graph_c0
AAAGTAACCTTCCTTGTTTCAACCATGGTTCTATTTAAACACCCAAAATTTA
CAAGGTAGAAAACAATGGAATATAACGACAGAGAAATAATTATTCCAACTT
AAACTCTGAAGAATACAAAAGATTATTCACAAGGTAGAGAACGATTACAGT
AAATTAGATTTGTGATCTCCATTGCAAGGTTGGAGATATTACCCATGTACAT
GTTCTTGCTTGTCAATGGTGACTTACGAACAGGCAATTCTTTGAATCCTTCT
TCACAGTCTCCAGGTCCCTGAAAAAACATACTAAGTCTTATATACAAGTCTC

CATAACAATCTTCACGGTATATCAACTTCAGAGCTTCATCAAGGTAAGCAAT
GCCTTGTTCATAGAGCTCATAACACATCTTAAGGCGCCACTTGGTCACCTCG
TCTTTGCAGTCCAGAAGAAGAAGAGTAATGTAAGAAGAAGTCATATTCCTC
TTGGCAATAGCCCTGTCGATGACGATACCCAGAATATCTCTCGAGTTCAAGC
TTGAATCACGGCTTCGAGGGTCAGCCTGAACTGAAGAGACGCACAATTTG
TTGAAAATAGTTCCATTGCACATTTTCTGAATCAAAGCTTTGCTGGGTAGTT
GTTTTTTGGCAACTTCAGTCGCCCCTGTGATTGTAGGATTGTTTAGAGAAAC
AGAGGAAGCAAAACATAAAAGGAATGCTAAACTAACCGTTAACAAAGCCA
TTTATAATTTTATATCTGAAACAGAGCACACAAATGATCTAAAAATGCTCTG
TTATG

>c58332.graph_c0
TAAATTATGATGTCATTTTACTCATTTGTTGTCAGAGCTTGTGTTTCCATTAA
TTATTATTGAGTAATAATGTGAACGTTAATAACAAGAACTGTGTATATTCATA
TACATAAGTACATTTTTTGGGTAATTTTGAATCCACTTTGTTCAGAGTTACAA
AACTAGGCAGTCCTCCCTAACTTTGATGAAGGCAGTTATTATACTGTTACAA
ACTCCACACATTTTCTTGGACAACTTTCTAGTTCATCATATCTCATATGTCAT
TCCAACCCTGAGAATCTTACCATGTGGAATTGCCATCATTTTCCCCTTTTGC
CTGAAGTTTTTCCTCAGAAAACTATAAGCATAGTTGACAACTATCTTCTTGA
AAAAGGAGGAATTCTGTTGTGCCACCACTTCTGCTTCTCCCAGGAGATAGA
CCACACCTCTGTCCTTTGCTCTCTGCACAAACTGCATCTCCTCTTCAGCTCC
CTGCAACGGCCTTGTGCTAATTCCATTGGATGAATTTGTTGACTTGGCCACA
TCAATCGACTGGTTGGATGCTGATGAACCACGAGTTGGGTTAGACTGTTGA
GATCGTTCCACATTGTGAACCAACGATGAACGAGATCCTTTTGCTTGTCCAT
CTTGCACTAGTATTGTCGAGTGCTCGAGGTTAACTGGATCAGCCATTTGCTC
AGTGGTTTCCCCTTCTTCGAGAATGAAATGTTCCTGGCGGATAAACTCTTTC
AACTGTTCCACTAGTTGGTTCTCAAACTCCTTGGGTTCTTCAATCTTGTCTT
TATATCCGTACCTTACAACGCATCTGAACATCCTGTAATCTCTTGGCTCGACC
TGTCGGAACAGATATCGCTCCTCCAGAGCTACTTTACTAATAGGAATGGACT
TAATTGAGACAAACACTAGAACTGAGTGGATGGATGGTATGTTGGCAATGA
AGTGAGGAAATATCGGAGGGATGCCTTGTACAAGTTCAGAATACAACAGTC
CTATTCCTGGCACCCGGTTTATTTCTGGGTTGGAGGCCAATTCTCTTATGAA
CCCACTAGAAACCTTGTTATTGAGCTCAAACATATACCTTTCTTTGTGTACG
TAATGCCATATCCCCATTACCACCATTAGGACAGATGCGAAGCCTAGAGGAA
GGTAACCACCCTGTGTGAATTTGTACAGGACTGCTGATAGATATGTGCCCTC
TACGGAAATAAAGACCACAAAGAAGAGAGCAACCAACCATATGCTCTTCTT
CCATATGACGAGCATTATAACAGTGGTCAAACTTGTTGAGATCACCATTACA
TACATTAACCACAACAACTCCATATGCATTGTCAATCTTTTCCACGGTCTTA
AATGCGGAAGTTACTATAACACAAGTAACCATAATCAAGTAATTGAGCTCTG
GTATGTAAACCTGACCGTCGTGCTTAGCAGAAGTATGAAATATCTTAACGTT
CGGGAAACAGCCTAGACTCACGGCCTGATACATAATCTGAAAAGCCCCTAA
TATCATAGTTTGGCAGGCTATAATTGCAGCTGCGACAGCCACAACGAATGT
GGGCCAATACAATGGATCTGCATACCAGTGGAATGTAAATAATAGAAAGTA
AAATTTTAAAGATTTTGAAGCTTCAAATCGTGACAAAAAACTATTTGAGTTA

ACAGTGTCCTGTAAGCTTTTACAACTATAAATCTGGACAATAAACTATTTGA
TTTTTTTAGTGTAAGATAGTGGGCCAACAGTGTATATCAAGCAAATACTCCT
CATCAGACTGACTGGTTAACAGATAGGTGTAATAAACGGGGATCAGATTTG
AACCCCTGATCTCTAAGACGCTAATACCAAATGCTAAAGTCATAAACACGT
GGTATGTGGATCAACAATGTATATCGAGTAACAAGGACTATCCTTTACAATA
GTACCTATTAAGAGAATTACCTGGAATCGAGTCATAAAAAGTATTTCCCACA
TTATTAGGGAATTTTGTGAGGTATGTAGCCTGCCCACTATATGCAAGTGCTA
ATGCAGGAAACACAACGCATAAGAAACTAATATACAAGTTTACAAAAGAG
AATAAATACACCAGTTCTTAGCGCCGGGAACCAGCTTGTTTTCAAAAAAAT
ATTGGTAAATGTCCAATGTATATCAGATCACCTGGACTGCTCGGACACTGAA
GTGACCAAGATCAGCAAACATGGCTTCCGTTCCTGTAATGCACATAACAAC
TCCACCAAGGGATATCCATCCCTTTTTACCATTTCTCTTGAAGTAATCTAAG
ATGTATATGGGATTGAAAGCACGCAATACACCCAAATCATATTTAAATAAGT
TGTGGAGACCAATGCCACTGATGAATAAAAACCATACACAGATGATGGGGG
CAAATGAGTATCCCACTCTATCGGTGCCAAATTGTTGAACAGAGAAGAGGA
TGATCAAGATTGCTACTGAAATTCCCACAACAGCATCTTCGCTTAGCGCCTT
GACACCGCTCACTGCGGAAAGGACAGAGATGCATGGAGTTAGGATTCCCT
CTCCAATCACCATGGAAGTACCAAGAATGGTGAAGAGGAAAAGCATCACT
TTGGCAGCCTTACTGTGCTCGAGCTTCCCTTTAATTTTTTCAGCTCGTCTCA
GATTATTTGATGGTATGTCTAGTCTGTAGTTGGATAGCTCCCTGTCCTCTGGC
TGCTGATTTGGAAGTAAGCTCACATTTGCATACCGGCTTAGCAAGGAATAC
AGGGCAAATGTTCCACCGACGCCATTGTCGTTGGCAGACAACACAATAAA
GACATATTTAATAAGGGGCACCAGCACCAGGGTATAGATTATGAGGGACAA
AACCCCAAGAATATCGTCTTTGTGGCCAATCTCATTGGGGAAAGTGCTGGC
GAACACGTACAGGGGAGAAGTTCCTATATCGCCATAGACAACTCCTATCGC
CTGAAAAGCTAAACTCAGCGTCGTCGTCCATTTTACCTTGGAGGAGGAATG
GGTTTGCGAATAAGTGAGTCTTCCAGCCTCCAAGTTGAGGGAGTCTACACG
GCGGAGTTTGGCCCATGACACCTTTCGCTCTTTCAGCTTGTTTTCGACTTCT
GTGGTGTCTAATCCTTCATTTGTATTAGTATCTGCCGCTTTCTCTGCCATTTTT
CTTATGAAATAAATCTGCTTCTGTTTCC

>c58361.graph_c0
GAAATTTATATGTTCTGTCCTTGGACCAACCAGATGTAGTTGCGTGTAACAG
TTGAGCATGCATATAGTTGTATATATATAATAACGACTACAACTTAAAATTGT
TATACTTCAAGTTCTTGATTTTACGCCCACGCAAAATCTGAAGTTAAAGAAA
TAATGGTTTCGAGGTGGTGGAAATTTATATGTTCTGTCCTTGCTGCTGAGAT
TATCTTCTTTCATGGCTTCTCCCGTTCAGAACCAGCCCCAAATTACACGTTT
GTCCATGAAGCAACATGGGCTCCTGCCACAGCCTCTTACGACTACATAATC
ATCGGTGGAGGAACTGCTGGTTGTCCGTTAGCCGCCACTCTCTCTCAGAAC
GCCACCGTCTTACTACTCGAAAGAGGCGGCTCGCCTTACGGAAACGACAA
CATCACCGAGATGGAATCTTTCGGCAACCCTCTAGCCGACCACTCTCCCTC
ATCGCCTTCCCAATTCTTTATCTCTAAAGATGGAGTCTTCAACTCAAGAGCA
CGTATACTAGGCGGCGGAACAGCCTTGAACGTTGGGTTCTATTCACGGGCT
GGTACGGAGTATGTTAAACAAATGGGTTGGGACCAAAGTTTGGTGAACAG

ATCGTTTGAATGGGTGGAAAAGAAGGTGGTGTTTGAGCCGCGGGTAACGC
CGTGGCAATCAGCAGTCAGAGATGGGCTGATTGAAGTGGGTGTGACACCG
TATAATGGGTTTACGTATGATCATTTACATGGGACTAAAGTTAGTGGCACAAT
ATTCGACCGGGAAGGTCATCGACACACTGCCGCTGATTTGCTTGAGTATGC
TATTCCAAGCAGGATTTCTGTTCTTTTGCATGCAAGAGTTCAAAGGATCTTG
TTTAGAATCAAAGGAAACAAAAGACCGAAAGCTATTGGAGTAGTATTCAA
AGATGAATTAGGAAATGAGCACATAGCGTATTTAATGAAGGGGTCAATGAA
CGAGATCATACTATCAGGCGGTGCAATTGGAAGTCCACAATTGTTGATGCTA
AGTGGTGTGGGCCCTAAACATCAACTTAAGGCCCACAACATCAGTCTAGTT
TTAGACCAACCCATGGTTGGCCAAGGGATGGCTGATAATCCAATGAATGCC
CTCTTCATTCCCTCTCCTCATCCGGTTGAAGTTTCCCTCGCTCAAGTTGTGG
GCATTACCCAATTTGATAGCTATATTGAAACAATGAGTGGGTTGGCCTTGAG
TTTTTTTTTAGCTCAAGATCAGAATGACCAGCCCTTTGCAGCCACTGTAAA
AGCTTTTGAAAGCATGAAAAATATTTCCAATGCAACTTACAATAGCGGAGT
TATAGCAGAGAAGTTCGTGGGACCTCTATCCACTGGCTACCTTGAGCTTCG
AAATAAGGACGTTGATGATAACCCTTACGTCACTTTTAACTATTTTAAAGAA
CCTGCGGACCTGCAAAGGTGTGTTAAAGCCATGAGAACAATTCTAAATGTT
ATAGATTCAAAAGCTTTCTCAAAATACCGGTATGAAAATACTTCCGTACAAG
TTCTTATTAACACGATGCTCAACTATCCATTGAACTTGCGGCCGAGACATGA
GATCAATGCATCCATTTCGTTGGAACAATTTTGTGTAGATACTGTGGTGACT
ATTTGGCATTACCATGGAGGTTGCCAAGTGGGCAAGGTTGTTGATGAAAAA
TATAAGGTTCTTGGTGTCGATGCACTTAGGGTTGTCGATGCATCTACGTTTAT
TAAATCCCCTGGAACTAATCCTCAAGCTACTATCATGATGCTCGGAAGGTAT
ATGGGAGAAAAGATATTGCAAGAGAGGTTATCAGATGGGAGAAAATAGAA
GATTATTTCATCTTTTCTATTATTTACTTTAATAAACTAAATTTAAAGTTATCA
ATAATTGAATATGTTGGCAATTGGTGGATGATAATTTGGTTACCTAAGAGTG
GTTTAAAGTTGGTTGATCTCAGTGGCCGCATATATTTAAAGTCATTCATAGCC
ATGTTTCAAGAGAGTTATGCTATAAATTATATGTGCAAGTTATTAATTACTCT
TCTCAGATTTTAAAATAAATGGACTTATATTTGG

>c57417.graph_c0
GAGATCAAGTTTCATCAATCTCTCCTTTTGTTAGCTTTGGGAAGAATCAACA
GATTGTCTTTTAACTTTGTAAAAATTCTAGAGATTCTACCCGCAACATTCAA
ATTAATATAAAGTTATATTTGCTGTCTCCATTCTTTATTTTGTGTTTAAGATGA
GAATTTAGTGCATACAATCATAATACACGTTACAGATAGTGCCTCTAAGTTG
TGAACTCGTGACCCAATAAAGATTTTCTAGTCAATGATTATTTTAGGTAAAT
AACATTATTTGTTTCTCCCTTTAAGCAGGGGAAGCTACTGAATTTCCAAGGT
CATTTTTCTTTGCAGTCTTGGAATTACAATCATCAAGATAATGCTGATAAAC
GTAGGAAACAAAACCCCACATGGCCAACAACATAGCAACCACCTTCACCC
CATCCATTTTATCATGGAAAAAAACCACAGCAAACACCGGAACCACAGGC
AAACCCAAAGTACTAATCACGTTAGAAAACAAAGAAGACACTTCAAAAAT
CAACCCAATCGCACCAATAGAAAAAATCTGCCAAGTCACTGCTGTCCAAAC
CAGATTCATCACATAAGACAATTTCCCCAGCTCATACCCCTCCATCTCCTTT
GTCAAACCCTTCCATTCCCCGCTCGCAAAGAGCCCAACTAGAATTGCGATA

GTGGCGACGAAATTCTGGTAGATAATCATGTCCAGAACCACTCTAAATGTTT
GCTTTTTCAGAACTTTTCGGAAACAGAACTGGGTTATAGAAAGCATTAAAG
CGTACCCAGCTGAGGCGCCAACGGTACATAAAAACCCAAGTATGTATTTCC
CTTTGGGTATTTTGGTTGAACCAGAAGAATCAGATTGGAAGACGAGGAGG
GTAGAGGATAGAGTGAGGAGGACTAAAGAGTTTATTATGAAAAATGTGAAT
TTTTGTGAATTGAGGAAGAAGGAGAAGAGCGCATTGAAAGCTAACTGGGA
TGCACAAATGAGTGAATACGTAGAGACTGGAAGGTATAATAACCCAATCGA
ATATAACATACAATCCGCTGCTAAGATTAAGCCTAAAGAGATATAAATCGAT
GCCACCTTCAAGGAAGAAGGTTTTTCTGAGTGAGTTGACTCGGAATGGAT
GGTTGGATTTTTGGATGGTGAGACGAAGAGACAGGGAAGAAGGATTGGGA
AGCCTGCGAGTTGTACAAGTGTTGCCATCCACTTGCTTTTCCCACCTTTGTC
GTAGTATAATCTTCCCAAGAGCGTCGCGACTGACTGGCCGGCGAGGACGA
AGACTGAGTATATTGCCATTCGGAGCCACCAATTGTAATTGAGTCCTGGAG
GTGGTGGTTGGCAGGTGATTTTTTCATGCTCGGGTAATTTAGCTTCTTTGGT
TTCCTGATCTATGATGTGGAGTTGTAGTTCTTGAGTTTCCCCCATGTCAATTG
TAAAAATACTCTCAAGTAAGATCAAGCTTAAAGCTGGAGTTTGCAGTGTGT
AAGAGAAAGCATGAAACGTATGAACGTTTTGAGAGAGAGAGAGACGTTTA
TCTCATGCGAAGCAAAGCATGGAATAGATGTGTAGAAATTGTTTAACATGC
ACCTTTGGCAGGTATGACTTGCGAGTCAAAAAAAAACTAACTGGATCCCAC
CGTCAACATCGATATTACATTACCACAGTATCCAACTAACCCATCACTAAAT
GCAGATTCAAATTTGTACCACGCCAAAACCCCCGCTCCTTCGCGCCGGTGT
CTACAATTCCATTTGCTTGTCCGTAGTTAATTGCTGTCACTATTGGAGGGCA
GGGACCAAATTATATTTTAATACATATCACTACAAATACATGGTCCAACTTTG
CCAACGTCGAGCCAAATTCCAGAAAGATAATTCTCAACCAACAAAAAAGA
ATTCCATTAATAATGAATCTTGAATTTGTTTGATACCGCCTACCGGACAACAT
ATATATGTCACAAACAATCTACCTTTCTCACCAATAAATTGGTTAAAAAGAA
AAAAGAAAAAAAAATCAAGTGTGACAATCTACAATGGCTGCTATACTCCCC
GCCTTCGACAAATTCAAGATGTATTATCAAATCCGATGCTGCAGCAATGGAG
GATGTTTTTCTCTCAAAAATCTACATTTTTAAAAGCAGCATCTTCCGTATCCT
TTTTATTGAACACTCCATCTACGTATATGTAAAAAATTTGGTATGTCCATGTC
CAACACAATATCTTCAAAACTCTTTTTGGCGTCTTGACTCATCAGGCCGTTG
TATGAGGGATCTGAAGAAGAATGAGTGAGATGCAATACAAGCAGAGAGAA
ATAGCAGCCCCCGCAATAAAATTACATAGAAGTCGTCGATTATCTTTTGTGG
GTACAAACACCGTCTTAAGTGAGTTGGTCAATTCAAAAAGGCGGAGAGAA
ACTAAAACATAAATTGCACTGGTGAGCATGAAGTTGAGCAATGGATATTCC
GGGATAAAAGAAAGAAGCCATTTGGGCTGACCATTAGGTACATTTGATCTC
AGCCAGATGTGGAACTGGCAGATATAGGTTTCCAAAGTTATCTTCCCAAGC
CATGCAAAGAGAGTCAAAGAGAAATTCCGAAGATGCTGTGTAAAATTTCG
CAAGCAAATATAAACAGTTATTGGAATCCATGATGTGTAGGGATGCAACTTG
TTGTAAGTAACCTTGTCCAGTTTGTAAATACATTCATACCACAAATAACCAA
CAAATACAGAAACAGTAACAATGCTTGTCTTAATTGTGAGTCTCCTCTTGGT
ATCAGATTCCTCTAATTTCTCCATCCACTTCTCAACATTGGGATGATAATAGG
CATATATCATTCCAATAATCCATATATATCGATCCAATCCAGATCGGAAATGCC
ACTCATGTAATCGCGGGAGATCTGGTTTCATAGGATCACTGTATCCTAATATG

AATGCTAAAGGACGCCAAAAAATCTCAAATACTCCAGGAATTTCCCAAATC
AAGATAACCACAAGAAAGCATGCAAGAATTTTCACAGCCATCACAGATCTT
ATTTCATTATACTTGTGAAAAATACCAAGAGCTCCATAGACCATAACAGTGA
AAAGCGTATGCATTGGACAAATATAGTACAGCATGTAGTCATTGTTCAGAAT
AATACAGCAAAATGCCACCAAGAAATTTAGACGCCACATCATCTGAGCAAA
GCGTGCAAGACTAAAATCCTTTCTGATATAATAATAAGAGAAATTTCCAAAC
CCAGTCATCCAGACGTATGCAGCAATAAAGACGCGTATTGCATTGTATATCT
CCGTTGCAGCAAAATAATGGTACATTAAGAACAGAACCTGCATCCATCCTTT
CCACTCT

>c58526.graph_c0
CACACTTTGATTTCCACATCCCGCGTTGCGTTTTTGATTTCTACATCCCACAT
TGGATGGGGTCACTTTTACAATTTTAAAAAATAAATGGTAATTGTCAAATTT
TATACAGCATAGTTGAGCTAATATATAATTCAGAAAAACTTCTTGGCGATGA
AAACCTTGCTCCCCGACACAAGCTTAGATGGCTGGGAATCATTATTCTTATC
CTTGAGGACAGTTGCCCAAATCGATACCATCATTACCGTCTGGAATATTTTG
GTCTCACTGCTCTAAAGCTGATCGCCACTGAGGAACGGAACGACCAATGTT
TCAGCATAATTATACTCTACTGCGTTGGCCGCATTCACGTACGCAATGTTGA
AGGAGTTGTCCCACGTGGGATAGTTTATCATGTTTCAAATGGACCAAGTCT
GTAATTCTTACAATTGAAGATGGGTTGTTCCTACTGTCTTGGACCAGGTCTG
ACTTATAACAGTTCTACTCTGCCAGACATTTTCAATTAAAATCATATGTTAGG
ACAACAGATCGGACATGAACCTTTATTCAACATTGGAAACGGTGGTCACCA
AGTATTCAAATATCAATAGTAAGGAAACGCATGATAGTTTAGCAGAGCTCCC
TGAAATCATGCATAAGTTGGCAAAAAAGACTGACGGTGATAATACCAAAAA
CGACTTGGAGGAGCTTCGCTCGGGTCTTCTTGAGGTTATTATTGAGAAGCT
TGGCAGCACAAGTTATGTTGCCAACGACTTAATAGTCGATGCCTGCGATGA
GATTATGGAATTGATGGTCGTGCTTTTGTCATCCACGGCGTAGGAGCAACCT
TTAATTATTAGTGGTTCTGCCCATTCGGTTATGGTCCTCTTAACTCCACCAGC
TAGTTTTTCTATTGGTTTTCTTCCCGTTAGAATCTCTAATAAAAGTATGCCAA
AACTGTAGACGTCGCAACTCTCGGATACCTTGCCCCACATGGCATATTCTGG
TGCCAGGTATCCCAAGGTGCCCTTAACACGGGTGGTCATGTGGCTAACGCC
TTCTGGGATTAACTTGGCAAACCCAAAATCAGCAACCAGTGGCTCAAAATT
TGAATCCAGTAGCACATTACTGGCCTTGATATCTCTATGAATGATGTGGGGT
GTCACCTCATGGTGCAAGTACAGTAGGCCTTCTGCGGAGCCGATTGCAACC
TTCATCCTCCTTTTCCAATCTAGTTGTACATCGGCTGCAAACTGGCCGTGGA
GATGAGAAAGTAAGCTGAGATTTGGCATGTAATCATAGACAATAAGCCGTT
GATCTGTTCCAGCGCAATAACCCCTAAGACCCAACAAATTTTTGTGTGCAA
CCCTTCCCAGAACTTCAACCTCTACCGCAAATTCCATTTCAGCTTTTAAATT
CATAGACTTCAACTTCTTCACAGCTATCTGCGCGCATATATATACATATTAAA
CAACCATTAAATTAATTAACTAAAGATATGATCCAATTAATAATAAGGGAATT
ATTTTGAACTGATTACCTGAAGACCATCACTCGTTTTTCCCCAATAAACACT
TCCAAACCCTCCTTCTCCAAGCTTGTTATCCTCGCTGAAACCATTAGTAGCC
GTATGCAATTCCTTGTACGTAAATATCCTCCAAGATGTGTTGCCACCACCGC
TCATTGTCACCCTACAAGCACACAATTAACAATAATTAAATAAATATTCATCT

TCTTAAACAATTTCATCTAGCTATATAGATTTTCAGTACGGCCTGGACATCAA
AATTAAGTCCCCCATCTTTGCTATACCCTTCATCAACCTTCTCTCCACCACA
GCAACTCAACGATGATCCCATTGTCACCCTACCACCTTCTCCGTTCTACTTT
CTTCTTTTATACTGGAATCATGCCCATTTGGGCAGTTTGCATCTCAGCCGAC
CTCGGCCGGCATTGCCTTTTTTATTTTAACGGTTATAACTAAGGAGTTGACT
CCATATGCTACACACGACGATGACACATGTACATAAAAATCAATAGCAACAC
CTCAATTAGCCTGATAGATGAACATCACCTTAATTAATCTAGATAGTAAATTC
CTCTTTCG

>c53143.graph_c0
AACTATCTCCAGTCTTATAATCACTAATGATGTCAGCCACTACCACTACTACA
GTTCCAAAGCGTTAGAAAATTGTATGCAAGTTCTCATCTTCAGTAATTGGAT
TCAGTTTACAAACTAATAAACATCTGATGCCTTTTATAATAAAGCACAATATT
AAACAAAAGAAACCACAATAATAAAAACATTATCCTTACTTTTAGTTCCAC
ATTGCACATCAAGTTACTATATTCCTAGAGAACTTGCTCGTCCACTTGAATAT
AACAAAAAGATTATCCCATTGTTCAATCCCCTTGATAAGGTAAGATAAACAT
ATCCTTTGATAAAAATAACTGATATTTATCCGCCGATGACACCAACAACAAT
ATTTGATAAGGTGATGGGGTCTAGGATTTGGCTACGCAAAAATGGTGAAAC
CGTGGTGGAGAGAAATGGATGATGTAAGACAGTTAAAATCTATAACATAAC
ATAAAAAATAAGAACAAAAATTTCAACGCCAACTGTAAATTTCAAAATCCA
ATAAATATTGTAACATTTAATACCAAAAAAATCTAATGCTGGAGAGGAAGAA
GATTGATTTCACTACTGGATCATTTGCACTTATCCAACCCTTTAGTGATCTCA
AATATTGAATGGTAGCTTAGGCATTTTACACCAAATTTGTTATGCAAGACCA
CTCTCTGGAGATTATGAAAGAAACAAAGAAACATGTAATTTGTTACAGAGG
CCACTCAAAAGATAGGATTCTGATAATCAAGATAGGATTGAACTATTAATTC
CTGATATGCAAGATAGAGAGAGAATATTTCCAAAGCTCATAAAAATGGATAA
AAGGAATCATTCAAAGAGAACATTGATCAATTCTTTAAGAATCTAACAGCA
TGATGATCCTTATCAATAAGAAAATTAGACTCGGATGATCCTCCTCCAGAAT
ATCTTACCTCTAAGATTACTGTGTAAACAAACTTGGTGGAGATTCAAATTGA
ACCAACGAAGGACTGAAATAAATCAGGCTAGAACCTTGACGCGTTGTAATA
CTTGATCGAGAAAATGAGATTGTGCTGTAGCTAACTGGTTATGATGATATAA
TTTTTATATAGAGACAGTGGGATTACAATTTTGAAAAAACAGAAGTGAAAA
GTTTGTGTAACCATAGATTTAAAAATACCAAACTGAAATCGGCAGCACACC
TCAGGAATCACAATCCGAAACACCTAGAATAAAAATAACAAAACCAATTAA
TTTAAGAAAATTCAAAGGGAATATCTTTATATATATATATAGAAATTGAAAAC
CAAAAATTCAATTCTTACCTTAATCGAAACCCTCAAATTACTGACAACAACC
CAGGATTTCATCTCGTCGAGCGAAGCCCTGAATCGAAACATCCTATAGAAA
TTGAAACCCTAGAAATTGAAAACTAAGAAAACTTACCCTATTTGTTCATCG
ATCAAAACCGAAGAAACCTCTGAGTTTCTTTTCAATTCACCGTCGTCGTTC
CTCGCTAAGTCACCGACCTTCTCTATAGTTTCTCTTCGGCCAAATCCCCTTT
AAGCACTCAAAATTCTTCATGATCTCTCGATCCCACTCTTGAGCTCTTGGCT
TTTTGAAACCCTCGATTCTCGCTGGTCTGTGTACGTCACCGGCATCAAATCA
GGGAGAAATACCCTTGGCCATCTTCCCTTTTGAGCTTTCTCGAGCTCTCTTG
GAACGCCTTATCTATTTCGACTCGTTATTATAGCTTCGCTTCTGTTCGGATGA

ATTGGCCTAGGATATTCGGATCCGTAGCACCCAATATTTCTTTATAAACATCT
TTGTCGACGTTTCCTAGGTCTTCAATAATTCATTGTTTCGTATTCTTTATGTG
CGCTTCGTTTTCATGCGGCGCTCTTGGGAATGGCCAGTTTTGAGATGAATG
AGTTTGGAATTAAGAGGAAAAGAAGAATCGAGAATAGGAGCGGGGGTGG
GAATATGGGAAGAATTAATTAGGAGAATTCGAGATTTGAGAAGTATCGAGA
ATGGGAGCGGGTGAAGAGAATGGGATTTAATGCAGAGCAAATGGGAAGAA
TTAATATGGAAGGTTCGAGATTTGAGAAGGATCGACACCCGAGAGGTATCA
TACTAAGAATTTAGTTTTATTTTTGAAGAAATCGAGTATTTTAGTAATCATTA
ATATGGAGGGTTCAAGATTTGAGA
