# Supplementary material for: Screening of Genes Related to Early and Late Flowering in Tree Peony Based on Bulked Segregant RNA Sequencing and Verification by Quantitative Real-Time PCR
Source: Molecules. 2018 Mar 19;23(3):689. doi: 10.3390/molecules23030689 (PMC6017042; doi:10.3390/molecules23030689)
Supplement: Supplementary file 1 [file molecules-23-00689-s001.zip › Supplementary Materials/1. Supplementary Materials/Supplementary Materials 2.pdf]

Table S1 Results of assembling sequencing data

Length Range	Contig	Transcript	Unigene
200-300	3,391,448(98.08%)	35,825(20.59%)	28,675(36.46%)
300-500	26,209(0.76%)	29,784(17.12%)	18,590(23.64%)
500-1000	20,612(0.60%)	37,489(21.55%)	14,495(18.43%)
1000-2000	13,563(0.39%)	42,831(24.62%)	10,892(13.85%)
2000+	5,900(0.17%)	28,031(16.11%)	5,993(7.62%)
Total Number	3,457,732	173,960	78,645
Total Length	204,735,802	192,528,500	57,589,618
N50 Length	49	1781	1282
Mean Length	59.211	1106.74	732.27

Table S2 291 genes with significant enrichment for the association loci

Chr	AllCount	AssoCount	P-value	FDR
c42942.graph_c0	4	4	0	0
c43262.graph_c0	4	4	0	0
c44957.graph_c0	4	4	0	0
c46352.graph_c0	4	4	0	0
c50822.graph_c0	11	11	0	0
c54685.graph_c0	14	10	0	0
c55121.graph_c0	9	8	0	0
c58332.graph_c0	9	7	0	0
c58361.graph_c0	13	12	0	0
c48021.graph_c0	6	5	1.63E-13	2.63E-10
c57940.graph_c0	17	7	2.03E-13	2.97E-10
c54876.graph_c0	17	6	2.19E-11	2.94E-08
c38215.graph_c0	7	4	4.58E-10	5.68E-07
c57417.graph_c0	16	5	1.23E-09	1.41E-06
c18087.graph_c0	4	3	2.99E-09	2.30E-06
c39158.graph_c0	4	3	2.99E-09	2.30E-06
c44829.graph_c0	4	3	2.99E-09	2.30E-06
c48861.graph_c0	4	3	2.99E-09	2.30E-06
c53204.graph_c0	9	4	2.72E-09	2.30E-06
c58096.graph_c0	4	3	2.99E-09	2.30E-06
c58556.graph_c1	9	4	2.72E-09	2.30E-06
c56705.graph_c0	21	5	8.04E-09	5.89E-06
c41733.graph_c0	5	3	1.49E-08	9.59E-06
c48857.graph_c0	5	3	1.49E-08	9.59E-06
c53813.graph_c0	5	3	1.49E-08	9.59E-06
c49024.graph_c0	12	4	1.68E-08	9.65E-06
c53060.graph_c0	12	4	1.68E-08	9.65E-06
c55738.graph_c0	12	4	1.68E-08	9.65E-06
c47080.graph_c0	6	3	4.44E-08	2.10E-05
c55633.graph_c0	14	4	4.19E-08	2.10E-05
c56403.graph_c0	6	3	4.44E-08	2.10E-05
c58526.graph_c0	14	4	4.19E-08	2.10E-05
c58710.graph_c0	6	3	4.44E-08	2.10E-05
c59042.graph_c1	6	3	4.44E-08	2.10E-05
c50926.graph_c0	7	3	1.03E-07	4.74E-05
c53143.graph_c0	8	3	2.05E-07	9.16E-05
c22844.graph_c0	3	2	4.05E-07	9.95E-05
c27895.graph_c0	3	2	4.05E-07	9.95E-05
c31028.graph_c0	3	2	4.05E-07	9.95E-05
c31880.graph_c0	3	2	4.05E-07	9.95E-05
c31918.graph_c0	3	2	4.05E-07	9.95E-05

c32101.graph_c0	3	2	4.05E-07	9.95E-05
c33415.graph_c0	3	2	4.05E-07	9.95E-05
c36201.graph_c0	3	2	4.05E-07	9.95E-05
c37008.graph_c1	3	2	4.05E-07	9.95E-05
c39180.graph_c0	3	2	4.05E-07	9.95E-05
c39738.graph_c0	3	2	4.05E-07	9.95E-05
c40112.graph_c0	3	2	4.05E-07	9.95E-05
c40724.graph_c0	3	2	4.05E-07	9.95E-05
c41177.graph_c0	3	2	4.05E-07	9.95E-05
c41266.graph_c0	3	2	4.05E-07	9.95E-05
c41848.graph_c0	3	2	4.05E-07	9.95E-05
c42177.graph_c0	3	2	4.05E-07	9.95E-05
c44913.graph_c0	3	2	4.05E-07	9.95E-05
c45192.graph_c0	3	2	4.05E-07	9.95E-05
c45808.graph_c0	3	2	4.05E-07	9.95E-05
c48601.graph_c0	3	2	4.05E-07	9.95E-05
c52684.graph_c0	3	2	4.05E-07	9.95E-05
c53420.graph_c0	9	3	3.66E-07	9.95E-05
c54328.graph_c0	3	2	4.05E-07	9.95E-05
c54369.graph_c0	3	2	4.05E-07	9.95E-05
c55385.graph_c0	3	2	4.05E-07	9.95E-05
c56440.graph_c0	21	4	4.08E-07	9.95E-05
c56666.graph_c0	3	2	4.05E-07	9.95E-05
c57254.graph_c0	3	2	4.05E-07	9.95E-05
c57801.graph_c1	3	2	4.05E-07	9.95E-05
c54393.graph_c0	11	3	9.48E-07	0.000228
c27548.graph_c0	4	2	1.61E-06	0.00026
c33977.graph_c0	4	2	1.61E-06	0.00026
c34439.graph_c0	4	2	1.61E-06	0.00026
c34723.graph_c0	4	2	1.61E-06	0.00026
c34936.graph_c0	4	2	1.61E-06	0.00026
c35343.graph_c0	4	2	1.61E-06	0.00026
c35966.graph_c0	4	2	1.61E-06	0.00026
c36215.graph_c0	4	2	1.61E-06	0.00026
c37960.graph_c0	4	2	1.61E-06	0.00026
c38258.graph_c0	4	2	1.61E-06	0.00026
c39612.graph_c0	4	2	1.61E-06	0.00026
c40196.graph_c0	4	2	1.61E-06	0.00026
c40205.graph_c0	4	2	1.61E-06	0.00026
c40564.graph_c0	4	2	1.61E-06	0.00026
c42508.graph_c0	4	2	1.61E-06	0.00026
c42840.graph_c0	4	2	1.61E-06	0.00026
c46112.graph_c0	4	2	1.61E-06	0.00026
c46153.graph_c0	4	2	1.61E-06	0.00026

c46503.graph_c0	4	2	1.61E-06	0.00026
c47084.graph_c0	4	2	1.61E-06	0.00026
c50230.graph_c0	4	2	1.61E-06	0.00026
c51992.graph_c0	4	2	1.61E-06	0.00026
c52467.graph_c0	4	2	1.61E-06	0.00026
c53043.graph_c0	4	2	1.61E-06	0.00026
c53670.graph_c0	4	2	1.61E-06	0.00026
c54850.graph_c0	4	2	1.61E-06	0.00026
c56412.graph_c0	4	2	1.61E-06	0.00026
c56436.graph_c0	4	2	1.61E-06	0.00026
c57149.graph_c1	12	3	1.41E-06	0.00026
c57468.graph_c0	4	2	1.61E-06	0.00026
c58811.graph_c0	27	4	1.56E-06	0.00026
c60694.graph_c0	4	2	1.61E-06	0.00026
c9420.graph_c0	4	2	1.61E-06	0.00026
c55848.graph_c0	13	3	2.03E-06	0.000317
c56456.graph_c0	13	3	2.03E-06	0.000317
c57975.graph_c1	13	3	2.03E-06	0.000317
c54243.graph_c0	14	3	2.83E-06	0.000438
c36503.graph_c0	5	2	4.01E-06	0.000577
c43623.graph_c0	5	2	4.01E-06	0.000577
c43817.graph_c0	5	2	4.01E-06	0.000577
c46274.graph_c0	5	2	4.01E-06	0.000577
c54200.graph_c0	5	2	4.01E-06	0.000577
c55972.graph_c1	5	2	4.01E-06	0.000577
c56577.graph_c0	5	2	4.01E-06	0.000577
c59086.graph_c0	5	2	4.01E-06	0.000577
c56246.graph_c0	16	3	5.08E-06	0.000724
c40556.graph_c0	6	2	7.97E-06	0.001027
c49083.graph_c0	6	2	7.97E-06	0.001027
c49692.graph_c0	6	2	7.97E-06	0.001027
c50698.graph_c0	6	2	7.97E-06	0.001027
c51543.graph_c0	6	2	7.97E-06	0.001027
c51897.graph_c0	6	2	7.97E-06	0.001027
c51930.graph_c0	6	2	7.97E-06	0.001027
c52396.graph_c0	6	2	7.97E-06	0.001027
c53220.graph_c0	6	2	7.97E-06	0.001027
c55278.graph_c0	6	2	7.97E-06	0.001027
c58080.graph_c0	6	2	7.97E-06	0.001027
c58184.graph_c2	6	2	7.97E-06	0.001027
c40743.graph_c0	7	2	1.39E-05	0.001656
c45422.graph_c0	7	2	1.39E-05	0.001656
c45499.graph_c0	7	2	1.39E-05	0.001656
c45835.graph_c0	7	2	1.39E-05	0.001656

c46351.graph_c0	7	2	1.39E-05	0.001656
c46642.graph_c0	7	2	1.39E-05	0.001656
c48760.graph_c0	7	2	1.39E-05	0.001656
c49366.graph_c0	7	2	1.39E-05	0.001656
c50750.graph_c0	7	2	1.39E-05	0.001656
c57175.graph_c0	7	2	1.39E-05	0.001656
c48597.graph_c0	8	2	2.21E-05	0.00247
c48989.graph_c0	8	2	2.21E-05	0.00247
c50301.graph_c0	8	2	2.21E-05	0.00247
c54338.graph_c0	8	2	2.21E-05	0.00247
c55796.graph_c1	8	2	2.21E-05	0.00247
c56056.graph_c0	8	2	2.21E-05	0.00247
c58512.graph_c0	8	2	2.21E-05	0.00247
c58516.graph_c0	8	2	2.21E-05	0.00247
c58585.graph_c0	8	2	2.21E-05	0.00247
c58996.graph_c0	24	3	2.83E-05	0.003141
c41422.graph_c0	9	2	3.29E-05	0.00349
c46873.graph_c0	9	2	3.29E-05	0.00349
c49475.graph_c0	9	2	3.29E-05	0.00349
c54820.graph_c0	9	2	3.29E-05	0.00349
c56081.graph_c0	9	2	3.29E-05	0.00349
c57165.graph_c0	9	2	3.29E-05	0.00349
c59006.graph_c0	9	2	3.29E-05	0.00349
c58001.graph_c0	26	3	3.93E-05	0.004112
c58740.graph_c0	26	3	3.93E-05	0.004112
c48060.graph_c0	10	2	4.68E-05	0.004801
c56384.graph_c1	10	2	4.68E-05	0.004801
c59087.graph_c0	10	2	4.68E-05	0.004801
c59079.graph_c0	54	4	5.17E-05	0.005275
c49285.graph_c0	11	2	6.40E-05	0.006323
c53284.graph_c0	11	2	6.40E-05	0.006323
c53829.graph_c0	11	2	6.40E-05	0.006323
c56345.graph_c0	11	2	6.40E-05	0.006323
c58873.graph_c0	11	2	6.40E-05	0.006323
c57362.graph_c0	12	2	8.48E-05	0.008282
c57873.graph_c0	12	2	8.48E-05	0.008282
c18390.graph_c0	3	1	0.000163646	0.009153
c20242.graph_c0	3	1	0.000163646	0.009153
c21244.graph_c0	3	1	0.000163646	0.009153
c22996.graph_c0	3	1	0.000163646	0.009153
c23385.graph_c0	3	1	0.000163646	0.009153
c24627.graph_c0	3	1	0.000163646	0.009153
c27123.graph_c0	3	1	0.000163646	0.009153
c28982.graph_c0	3	1	0.000163646	0.009153

c30026.graph_c0	3	1	0.000163646	0.009153
c30067.graph_c0	3	1	0.000163646	0.009153
c30277.graph_c0	3	1	0.000163646	0.009153
c31037.graph_c0	3	1	0.000163646	0.009153
c32193.graph_c0	3	1	0.000163646	0.009153
c34617.graph_c0	3	1	0.000163646	0.009153
c35512.graph_c0	3	1	0.000163646	0.009153
c35765.graph_c0	3	1	0.000163646	0.009153
c36624.graph_c0	3	1	0.000163646	0.009153
c36807.graph_c0	3	1	0.000163646	0.009153
c36999.graph_c0	3	1	0.000163646	0.009153
c37171.graph_c0	3	1	0.000163646	0.009153
c37472.graph_c0	3	1	0.000163646	0.009153
c37547.graph_c0	3	1	0.000163646	0.009153
c37820.graph_c0	3	1	0.000163646	0.009153
c38048.graph_c0	3	1	0.000163646	0.009153
c38084.graph_c0	3	1	0.000163646	0.009153
c38623.graph_c0	3	1	0.000163646	0.009153
c38661.graph_c0	3	1	0.000163646	0.009153
c38691.graph_c0	3	1	0.000163646	0.009153
c38771.graph_c0	3	1	0.000163646	0.009153
c38961.graph_c0	3	1	0.000163646	0.009153
c39162.graph_c0	3	1	0.000163646	0.009153
c39337.graph_c0	3	1	0.000163646	0.009153
c39433.graph_c1	3	1	0.000163646	0.009153
c39683.graph_c0	3	1	0.000163646	0.009153
c40002.graph_c0	3	1	0.000163646	0.009153
c40342.graph_c0	3	1	0.000163646	0.009153
c40574.graph_c0	3	1	0.000163646	0.009153
c40775.graph_c0	3	1	0.000163646	0.009153
c40850.graph_c0	3	1	0.000163646	0.009153
c41171.graph_c0	3	1	0.000163646	0.009153
c41521.graph_c0	3	1	0.000163646	0.009153
c41998.graph_c0	3	1	0.000163646	0.009153
c42204.graph_c0	3	1	0.000163646	0.009153
c42215.graph_c0	3	1	0.000163646	0.009153
c42291.graph_c0	3	1	0.000163646	0.009153
c42334.graph_c0	3	1	0.000163646	0.009153
c42364.graph_c0	3	1	0.000163646	0.009153
c42490.graph_c0	3	1	0.000163646	0.009153
c42660.graph_c0	3	1	0.000163646	0.009153
c42677.graph_c0	3	1	0.000163646	0.009153
c42879.graph_c0	3	1	0.000163646	0.009153
c42917.graph_c0	3	1	0.000163646	0.009153

c43111.graph_c0	3	1	0.000163646	0.009153
c43152.graph_c0	3	1	0.000163646	0.009153
c43219.graph_c0	3	1	0.000163646	0.009153
c43513.graph_c0	3	1	0.000163646	0.009153
c43840.graph_c0	3	1	0.000163646	0.009153
c43964.graph_c0	3	1	0.000163646	0.009153
c44357.graph_c0	3	1	0.000163646	0.009153
c44448.graph_c0	3	1	0.000163646	0.009153
c44532.graph_c0	3	1	0.000163646	0.009153
c44605.graph_c0	3	1	0.000163646	0.009153
c44649.graph_c0	3	1	0.000163646	0.009153
c44715.graph_c0	3	1	0.000163646	0.009153
c44932.graph_c0	3	1	0.000163646	0.009153
c45144.graph_c0	3	1	0.000163646	0.009153
c45849.graph_c0	13	2	0.000109671	0.009153
c45986.graph_c0	3	1	0.000163646	0.009153
c45997.graph_c0	3	1	0.000163646	0.009153
c46357.graph_c0	3	1	0.000163646	0.009153
c46845.graph_c0	3	1	0.000163646	0.009153
c46880.graph_c0	3	1	0.000163646	0.009153
c47117.graph_c0	3	1	0.000163646	0.009153
c48109.graph_c0	3	1	0.000163646	0.009153
c48219.graph_c0	3	1	0.000163646	0.009153
c48340.graph_c0	3	1	0.000163646	0.009153
c48393.graph_c0	3	1	0.000163646	0.009153
c48434.graph_c0	3	1	0.000163646	0.009153
c48626.graph_c0	3	1	0.000163646	0.009153
c48717.graph_c1	3	1	0.000163646	0.009153
c49271.graph_c0	3	1	0.000163646	0.009153
c50032.graph_c0	3	1	0.000163646	0.009153
c50045.graph_c0	3	1	0.000163646	0.009153
c50544.graph_c0	3	1	0.000163646	0.009153
c51023.graph_c0	3	1	0.000163646	0.009153
c52104.graph_c0	13	2	0.000109671	0.009153
c52363.graph_c0	3	1	0.000163646	0.009153
c52407.graph_c0	3	1	0.000163646	0.009153
c52603.graph_c0	3	1	0.000163646	0.009153
c52646.graph_c0	3	1	0.000163646	0.009153
c52729.graph_c0	3	1	0.000163646	0.009153
c52930.graph_c0	3	1	0.000163646	0.009153
c52995.graph_c0	3	1	0.000163646	0.009153
c53106.graph_c0	3	1	0.000163646	0.009153
c53158.graph_c0	14	2	0.00013881	0.009153
c53189.graph_c0	3	1	0.000163646	0.009153

c53386.graph_c0	14	2	0.00013881	0.009153
c53666.graph_c0	3	1	0.000163646	0.009153
c53676.graph_c0	3	1	0.000163646	0.009153
c54007.graph_c0	14	2	0.00013881	0.009153
c54479.graph_c0	13	2	0.000109671	0.009153
c54656.graph_c0	3	1	0.000163646	0.009153
c54888.graph_c0	14	2	0.00013881	0.009153
c54996.graph_c0	3	1	0.000163646	0.009153
c55089.graph_c0	3	1	0.000163646	0.009153
c55766.graph_c0	3	1	0.000163646	0.009153
c56101.graph_c0	3	1	0.000163646	0.009153
c56164.graph_c0	3	1	0.000163646	0.009153
c56465.graph_c0	14	2	0.00013881	0.009153
c56661.graph_c0	3	1	0.000163646	0.009153
c57014.graph_c1	3	1	0.000163646	0.009153
c57316.graph_c0	14	2	0.00013881	0.009153
c57550.graph_c0	14	2	0.00013881	0.009153
c57593.graph_c0	3	1	0.000163646	0.009153
c57932.graph_c1	13	2	0.000109671	0.009153
c58005.graph_c0	13	2	0.000109671	0.009153
c58075.graph_c0	3	1	0.000163646	0.009153
c58151.graph_c0	3	1	0.000163646	0.009153
c58312.graph_c0	13	2	0.000109671	0.009153
c58329.graph_c0	3	1	0.000163646	0.009153
c59042.graph_c0	3	1	0.000163646	0.009153
c61079.graph_c0	3	1	0.000163646	0.009153
c63409.graph_c0	3	1	0.000163646	0.009153
c57190.graph_c0	15	2	0.000172555	0.009552
c58949.graph_c0	15	2	0.000172555	0.009552
c59000.graph_c0	15	2	0.000172555	0.009552

Table S3. The primer sequence of qRT-PCR

Rrimers	Sequences	Size (bp)
*q42942*F	5' GGCTTTCCCTTCTACGGCTAT 3'	177
q*42942*R	5' ATTGAACACTGTTGTGGCACC 3'	
q*46352*F	5' CCACTTGGTCACCTCGTCTT 3'	92
q*46352*R	5' TCGTCATCGACAGGGCTATT 3'	
q58332F	5' GAATGGGTTTGCGAATAAGTG 3'	93
q58332R	5' GCTGAAAGAGCGAAAGGTGT 3'	
q58361F	5' CGTGGCAATCAGCAGTCAGA 3'	147
q58361R	5' CAAGCAAATCAGCGGCAGT 3'	
q54876F	5' GTTATCTGTAAATCTAGGCTGGGG 3'	129
q54876R	5' TTTGCTGGTGGTGCTCTACAT 3'	
q57417F	5' GCGATAGTGGCGACGAAA 3'	222
q57417R	5' TCTATCCTCTACCCTCCTCGTC 3'	
q55633F	5' CTTCTCATCCTTCCACTTTGTCTTA 3'	120
q55633R	5' AAAACCGACAAGGAATGGGAG 3'	
q58526F	5' ATTCAACATTGGAAACGGTGG 3'	236
q58526R	5' AATCTCATCGCAGGCATCG 3'	
q53143F	5' GGAATCACAATCCGAAACACC 3'	171
q53143R	5' CGACGAGATGAAATCCTGGG 3'	
qActinF	5' GGTCTATTCTTGCTTCCCTCAG 3'	
qActinR	5'GAACTCACTATCAAACCCTCCAG3'	

Table S4 The flowering time of parental and twenty early-flowering and late-flowering of F_1 population strains.

Number of strain	First-flowering dates (Month. Date)	Last-flowering dates (Month. Date)	Blooming period (Days)
male parent	4.17	4.23	7
female parent	4.11	4.21	11
7-26	4.3	4.12	10
7-29	4.3	4.12	10
7-30	4.3	4.12	10
7-46	4.3	4.12	10
7-50	4.3	4.12	15
7-58	4.3	4.14	12
9-13	4.3	4.13	11
9-26	4.3	4.12	10
7-22	4.4	4.13	10
7-33	4.4	4.12	9
7-35	4.4	4.12	9
9-25	4.4	4.12	9
7-15	4.5	4.17	13
7-28	4.5	4.17	13
7-52	4.5	4.12	8
8-23	4.5	4.17	13
8-60	4.5	4.17	13
8-62	4.5	4.10	6
8-30	4.6	4.20	15
8-4	4.6	4.14	9
8-2	4.8	4.14	7
8-20	4.8	4.19	12
8-21	4.8	4.15	8
8-24	4.8	4.19	12
8-33	4.8	4.14	7
8-50	4.8	4.17	10
8-59	4.8	4.17	10
8-63	4.8	4.20	13
9-3	4.8	4.14	7
7-11	4.9	4.17	9
7-18	4.9	4.17	9
7-38	4.9	4.17	9
7-51	4.9	4.17	9
7-59	4.9	4.17	9
8-70	4.9	4.19	11
7-31	4.10	4.17	8

8-22	4.10	4.20	11
8-28	4.10	4.20	11
8-53	4.10	4.13	4
8-47	4.14	4.17	4
