# Supplementary figures and images for: Screening of Genes Related to Early and Late Flowering in Tree Peony Based on Bulked Segregant RNA Sequencing and Verification by Quantitative Real-Time PCR
Source: Molecules. 2018 Mar 19;23(3):689. doi: 10.3390/molecules23030689 (PMC6017042; doi:10.3390/molecules23030689)

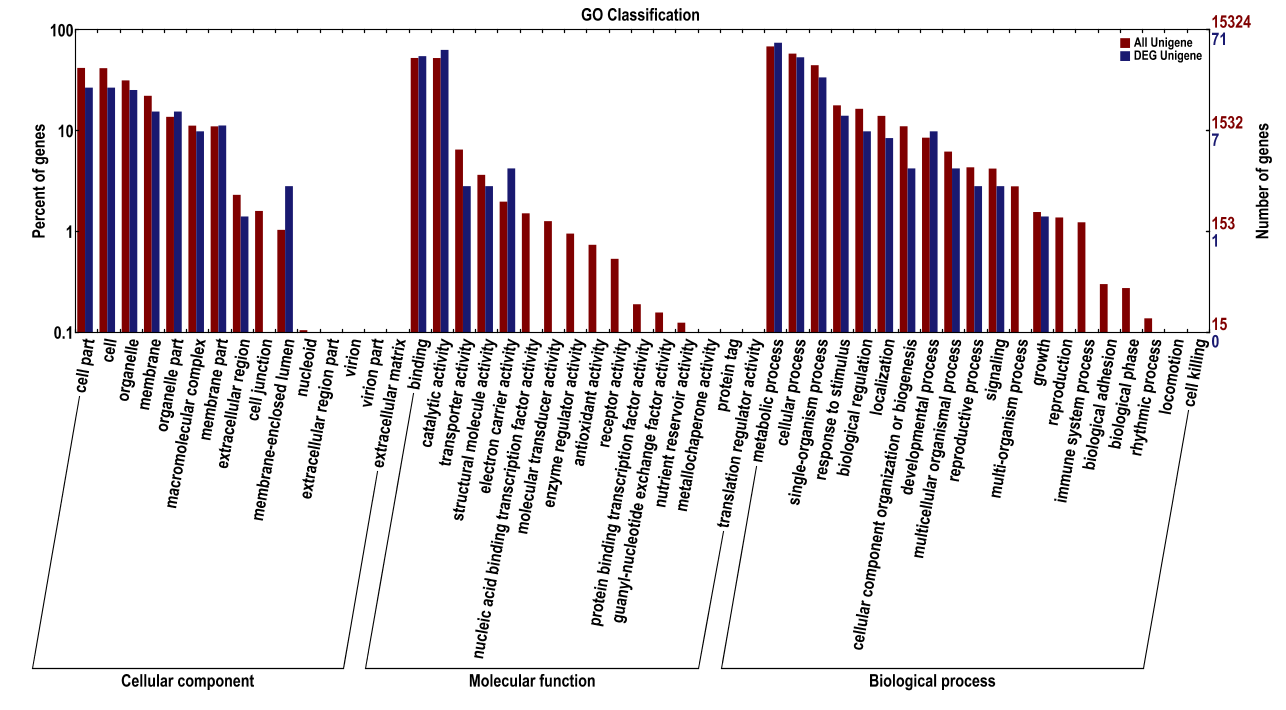

Supplement: Supplementary file 1 [file molecules-23-00689-s001.zip › Supplementary Materials/2. Figures/Figure S2.png]

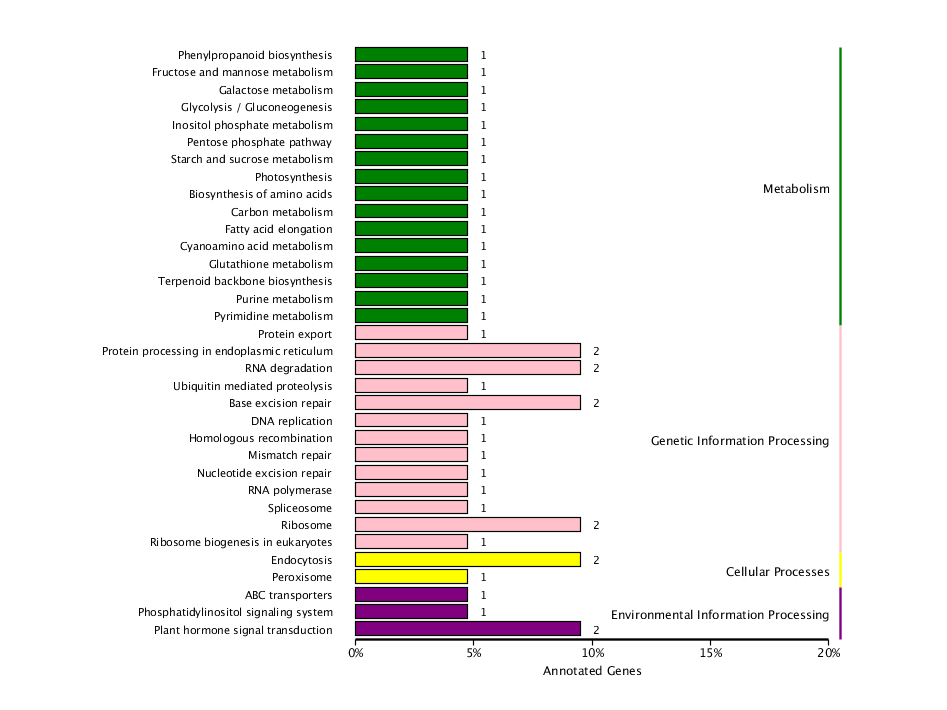

Supplement: Supplementary file 1 [file molecules-23-00689-s001.zip › Supplementary Materials/2. Figures/Figure S3.png]

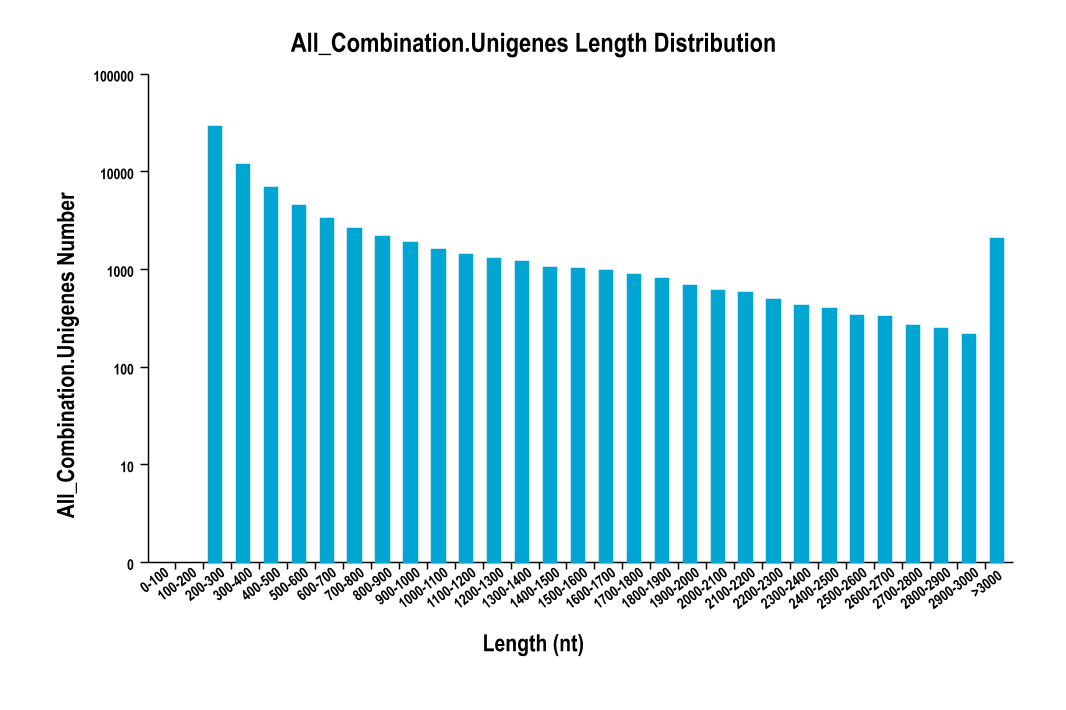

Supplement: Supplementary file 1 [file molecules-23-00689-s001.zip › Supplementary Materials/2. Figures/Figure. S1.jpg]
